# Purkinje cell microzones mediate distinct kinematics of a single movement

**DOI:** 10.1038/s41467-023-40111-5

**Published:** 2023-07-19

**Authors:** François G. C. Blot, Joshua J. White, Amy van Hattem, Licia Scotti, Vaishnavi Balaji, Youri Adolfs, R. Jeroen Pasterkamp, Chris I. De Zeeuw, Martijn Schonewille

**Affiliations:** 1 Department of Neuroscience, Erasmus MC, Rotterdam, The Netherlands; 2 Department of Translational Neuroscience, University Medical Center Utrecht, Brain Center, Utrecht University, Utrecht, The Netherlands; 3 Netherlands Institute for Neuroscience, Royal Netherlands Academy of Arts and Sciences, Amsterdam, Netherlands

**Keywords:** Cerebellum, Reflexes, Neural circuits

## Abstract

The classification of neuronal subpopulations has significantly advanced, yet its relevance for behavior remains unclear. The highly organized flocculus of the cerebellum, known to fine-tune multi-axial eye movements, is an ideal substrate for the study of potential functions of neuronal subpopulations. Here, we demonstrate that its recently identified subpopulations of 9+ and 9- Purkinje cells exhibit an intermediate Aldolase C expression and electrophysiological profile, providing evidence for a graded continuum of intrinsic properties among PC subpopulations. By identifying and utilizing two Cre-lines that genetically target these floccular domains, we show with high spatial specificity that these subpopulations of Purkinje cells participate in separate micromodules with topographically organized connections. Finally, optogenetic excitation of the respective subpopulations results in movements around the same axis in space, yet with distinct kinematic profiles. These results indicate that Purkinje cell subpopulations integrate in discrete circuits and mediate particular parameters of single movements.

## Abstract (web summary)

Purkinje cells form a diverse population, but how diversity contributes to cerebellar behavior is not fully understood. Here, the authors reveal how nuances in molecular signatures correlate with electrophysiological, anatomical, and ultimately functional differences among Purkinje cell subpopulations.

## Introduction

Neuronal populations are classically defined based on morphology, lineage, response properties, connectivity and/or location within the brain. Recent technological and methodological developments have revealed further divisions into subpopulations, primarily characterized by distinct molecular signatures and intrinsic electrophysiological properties^1,2^. However, little is known about the role of neuronal subpopulations in behavioral output^3,4^. The cerebellum is ideal for the study of the functional impact of neuronal subpopulations because of both its well-organized anatomy and its direct association with motor output^5^. In the cerebellar cortex, Purkinje cell (PC) neuronal diversity is highlighted by the differential expression of Aldolase C (AldoC) protein, which establishes AldoC-positive (AldoC + ) and negative (AldoC−) subpopulations^6^. The cerebellar AldoC map largely reveals the PC component of cerebellar modules^7,8^. Cerebellar modules are formed by parasagittal microzones of PCs that receive climbing fiber input from a specific region of the inferior olive and converge onto a specific area of the cerebellar and/or vestibular nuclei^9^. However, the AldoC map reflects only part of the empirical diversity of PC subpopulations^1^, as seen by the nuanced range of AldoC expression levels along cerebellar parasagittal domains (e.g., 3+, 3b+, e1−, e1+, e2+)^10,11^ and the expression profile of molecular markers, such as Nfh^12^, Hsp25^13^ or TrpC3^14^ that only partially overlap with AldoC domains. To be able to address the behavioral relevance of PC diversity, additional markers and tools are necessary to visualize and selectively manipulate PC subpopulations.

The modular organization of the cerebellum is fundamental to its structure^6^. It is mirrored by input and output circuitry^9^ as well as intrinsic firing properties^15^ and learning rules^16,17^. Differential connectivity between microzones of PCs suggests that they are specified for discrete behavioral control^18,19^. However, multiple microzones, including combinations of AldoC+ and AldoC− subpopulations, can relate to the same muscle group^20^ or respond to the same specific behavior^21^. In order to gain a complete understanding of cerebellar function, it is necessary to determine how discrete cerebellar microzones influence behavior. The described PC diversity suggests that PC microzones are physiologically tuned by differential gene expression patterns to the specific demands of the behavioral circuitry in which they participate^22,23^. Strategies have been developed to indirectly interrogate distinct microcircuits by targeting subdivisions of either cerebellar nuclei^24^ or inferior olive nuclei^25^. However, genetic targeting of PC microzones has proven challenging. To date, no genetic tool offers the refined spatial resolution to selectively monitor and/or manipulate individual PC subpopulations. Here, we aim to identify and apply new genetic tools to uncover the functional potential of individual cerebellar microzones.

We focus our investigation on the flocculus to study structure-function relations for multiple reasons. First, the flocculus is a sensorimotor structure that integrates vestibular, visual, visuo-motor and proprioceptive sensory inputs to drive ocular movements to compensate for involuntary head motions and retinal slip^26,27^; this complex behavior can be readily measured with high-resolution video recordings of the eye^28^. Second, the flocculus can be divided into five functional zones defined by olivocerebellar climbing fiber input. PC simple spikes (SSs) and complex spikes (CSs) modulate in response to optokinetic stimulation about either the earth-vertical axis (VA, zones 2 and 4) or the horizontal axis that runs through the posterior semicircular canal on the ipsilateral side (HA, zones 1 and 3), or not to visual stimulation at all (zone C2)^28^. Third, the flocculus was considered to contain only an AldoC+ subpopulation^5^, but analysis of the AldoC-Venus knock-in reporter mouse^10^ has parceled the flocculus into two domains, the AldoC+ caudal aspect (9+) and AldoC− rostral aspect (9−), for which possible differences in physiology, anatomy, or behavioral output remain currently unknown. Finally, non-selective optogenetic stimulation of PCs in the flocculus can drive eye movements with a commonly mixed kinematic profile in various directions^29,30^. This raises the possibility that, if one had access to microzonal-specific manipulation, one might in principle be able to uncover the role of microzones in control of components of movement. Thus, the diversity of floccular microzones together with the fact that it is accessible for optogenetic stimulation and controls a behavior that can be readily measured in 3D-space at a high temporal resolution^27,31–33^ offers the perfect opportunity to determine the behavioral relevance of discrete neuronal subpopulations.

Taking advantage of the uncovered 9+ and 9− regions, we first identified optimal molecular markers to study this diversity and then determined the physiological differences among these floccular PC subpopulations. This analysis allowed us to identify two mouse models for selective genetic targeting of PC subpopulations in the flocculus for anatomical tracing and analysis of behavioral consequences of selective activation. With the use of these new tools, we provide evidence that PC subpopulations mediate distinct kinematic properties of a movement and that molecular diversity and connectivity are tuned to the specific needs of the behavior involved.

## Results

### PC subpopulations in the flocculus exhibit complex molecular profiles

**Fig. 1:**
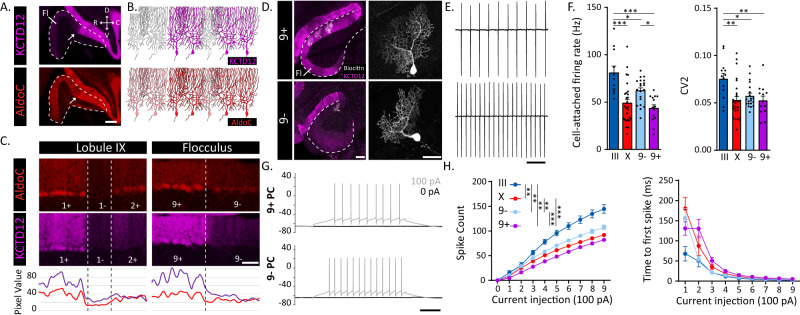
KCTD12 identifies physiologically distinct subpopulations of 9+ and 9- PCs in the flocculus. **A** Sagittal sections of the flocculus (white dashed line) immunolabeled for KCTD12 (purple) and AldoC (red). White arrows indicate the KCTD12 boundary identified with immunostaining. Scale bar = 200 µm. **B** Schematic representation of differential protein expression among floccular Purkinje cell (PC) subpopulations. **C** Top: Immunolabelling of KCTD12 and AldoC in lobule IX (coronal) and the flocculus (sagittal) labeling the cerebellar modules. Bottom: Relative signal intensity of KCTD12 (purple curve) and AldoC (red curve) expression in the PC layer for the top region. Scale bar = 50 µm. **D** Example of biotin-filled PCs in different regions of the flocculus. Scale bar left panels = 200 µm, right panels = 100 µm. **E** Example cell-attached recordings from 9+ (top) and 9− (bottom) PCs. Scale bar = 100 ms. **F** Summary data of firing rate and CV2 for PCs in Lobule III, Lobule X, 9- flocculus and 9+ flocculus. *n* = 14, 35, 20, and 14 cells, respectively. **G** Example traces from current injections steps. Black trace indicates 0 pA and gray trace +100 pA current injections around holding current. Scale bar = 100 ms. **H** Summary data of current injection steps for PCs in Lobule III, Lobule X, 9- flocculus and 9+ flocculus. *n* = 20, 51, 32, and 36 cells, respectively. Left graph displays the average number of spikes in response to a 500 ms current pulse ranging from +100 to +900 pA. Right graph displays the average time to the first spike at each of the current injection steps. Fl flocculus, C caudal, R rostral, D dorsal, V ventral. ***: *p* < 0.001, **: *p* < 0.01; *: *p* < 0.05 based on one-way ANOVA with multiple comparisons (see Supplementary Table 1). All data are presented as mean values ± SEM.

To identify markers that clearly differentiate PC subpopulations in the flocculus, we first searched the Allen Brain Atlas in situ hybridization database and found several candidate genes (Fig. S1). The potassium channel tetramerization domain containing 12 (KCTD12) protein^34^ exhibits a high contrast of expression that optimally identifies floccular PC subpopulations (Fig. S1). Immunohistochemical staining confirmed that KCTD12 divides the flocculus roughly in half along the rostro-caudal axis (Figs. 1A, S1), which matches the previously described 9+ (caudal) and 9− (rostral) domains (Fig. S2). In comparison, the difference between AldoC expression levels is more nuanced (Fig. 1C, Fig. S2B), complicating the division into AldoC+ and AldoC− domains in the flocculus (Fig. 1B). Similar to AldoC expression, PLCβ4 shows a subtle disparity in expression between both domains (Fig. S2C). For these two commonly used markers, the distinction in protein levels is low, making the contrast between PC subpopulations more dependent on the quality of the histological process. Nfh expression confirms that other known PC stripe markers can also discriminate between the 9+ and 9− subpopulations (Fig. S2D). To date, only Hsp25 expression was thoroughly described in the flocculus^35^. However, Hsp25 expression does not completely match the 9+/9− map, and Hsp25-immunoreactive PCs are a small subdivision of 9+ PCs (Fig. S2E). All together, these data demonstrate the presence of genetically delineated PC subpopulations in the flocculus, with the 9+ and 9− groups as the ideal targets for cell-specific manipulation.

### 9+ and 9- PCs exhibit distinct intrinsic physiological properties

PC subpopulations divided based on AldoC expression exhibit differential electrophysiological properties both in vivo and ex vivo^15^. To determine if similar differences exist in floccular subpopulations, we recorded PCs juxtacellularly and whole-cell in ex vivo cerebellar slices. Data from recorded cells were sorted based on histological identification of KCTD12 expression (Fig. 1D and Methods). We found that 9+ PCs fire at a lower rate (*p* = 0.0294) and are less excitable (*p* < 0.0001) than 9- PCs, similar to AldoC+ PCs relative to AldoC- PCs^15^. In their firing rate (*p* = 0.8093), regularity (>0.999) and excitability (*p* = 0.1202), 9+ PCs (KCTD12+/AldoC+) are similar to AldoC+ PCs found in posterior lobules. However, while clearly distinct from AldoC+ and 9+ PCs, 9− floccular PCs (KCTD12−/AldoC+) fire at a significantly lower frequency (*p* = 0.0124), are more regular (*p* = 0.0301), and less excitable (*p* < 0.0001) than KCTD12−/AldoC− PCs recorded in the anterior vermis (Fig. 1E–H). Together with the protein expression profiles, the electrophysiological properties of 9− PCs in the flocculus define them as a subpopulation with features between AldoC+ and AldoC− PCs.

### Identification of Cre lines to genetically target PC subpopulations

**Fig. 2:**
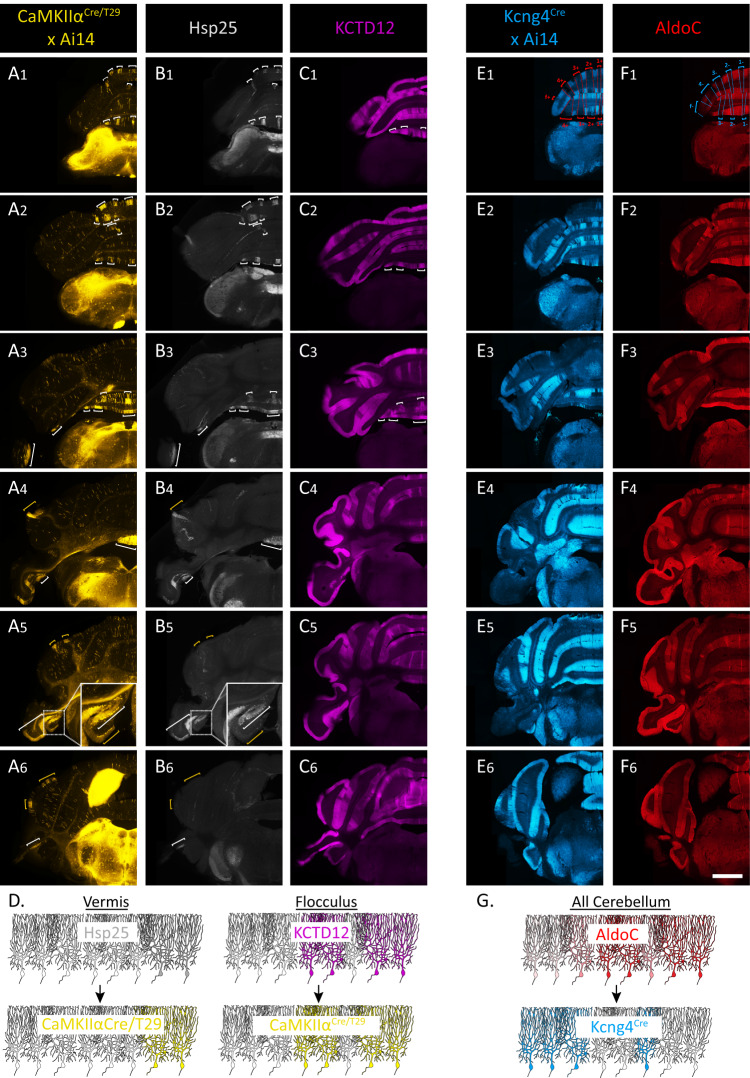
Novel mouse models *CaMKIIα*^*Cre/T29*^ (transgenic) and *Kcng4*^*Cre*^ (knock-in/knock-out) to discriminate PC subpopulations. **A**–**C** and **E**,**F** Serial transverse sections of the cerebellum of *CaMKIIα*^*Cre/T29*^*;Ai14* (**A**) and *Kcng4*^*Cre*^*;Ai14* (**E**) mouse immunolabeled for Hsp25 (**B**), KCTD12 (**C**), and AldoC (**F**). White brackets indicate the Hsp25 domains (**B**) and the corresponding TdTomato+ domains in the *CaMKIIα*^*Cre/T29*^*;Ai14* (**A**), as well as KCTD12+ domains (**C**). Yellow brackets indicate the TdTomato+ domains from the *CaMKIIα*^*Cre/T29*^*;Ai14*, which do not correspond to any Hsp25+ domains, with a zoom on the flocculus (A_5_ and B_5_) to highlight that in this particular region, *CaMKIIα*^*Cre/T29*^ expression extends beyond Hsp25 and matches KCTD12 expression (expression pattern confirmed in *n* = 4 *CaMKIIα*^*Cre/T29*^*;Ai14* mice). Blue and red brackets and dotted lines show the complementation of TdTomato +, from the *Kcng4*^*Cre*^*;Ai14* and the AldoC+ populations (E_1_ and F_1_). Scale bar = 1 mm (applies to all images). **D** Schematic representation of Cre expression in the *CaMKIIα*^*Cre/T29*^ relative to Hsp25 and KCTD12 expression profiles. **G** Schematic representation of Cre expression in the Kcng4^Cre^ relative to AldoC expression profile.

In order to genetically target the PC subpopulations in the flocculus, we screened the Allen Brain Atlas and GenSat databases and identified two genetically-engineered mouse lines, the transgenic *CaMKIIα*^*Cre/T29*^ line and the knock-in *Kcng4*^*Cre*^ line. Crossed with a reporter line (*Ai14*), *CaMKIIα*^*Cre/T29*^ revealed organized clusters of PCs in lobule VI, ventral lobule IX and lobule X, lobule simplex, anterior Crus1, ventral paraflocculus, and flocculus (Fig. 2A–D), with varying degree of overlap with known markers. In ventral lobule IX and lobule X, *CaMKIIα*^*Cre/T29*^ + PC clusters match with the Hsp25-immunoreactive (Hsp25 + ) PCs and KCTD12+ domains. In the paraflocculus, Cre expression correlates with Hsp25+ domains but not KCTD12+ domains. In lobule simplex and anterior Crus1, *CaMKIIα*^*Cre/T29*^ + PC clusters did not correspond to either Hsp25+ or KCTD12+ domains. Finally, in the flocculus we observed that *CaMKIIα*^*Cre/T29*^ + PCs perfectly match with the KCTD12+ domain, which represent a larger PC group than the Hsp25+ PCs (Fig. 2D). In addition, *CaMKIIα*^*Cre/T29*^ drives expression in PCs sparsely distributed throughout the cerebellum. These data were surprising given that previous immunohistological studies on *CaMKIIα* expression in the cerebellum reported a homogeneous expression of the protein in all PCs^36,37^. These diverging results suggest possible artifactual expression in this transgenic line due to the transgene construct, insertion and/or copy number. However, the Cre expression pattern was roughly consistent in all biological replicates across all regions, demonstrating a stable and reproducible genetic targeting of specific PC subpopulations. In *Kcng4*^*Cre*^*;Ai14* mice, we observed expression of TdTomato in a large group of PCs distributed in domains primarily complementing the AldoC expression pattern (Fig. 2E–G). In the flocculus, we observed a differential expression of TdTomato in PCs between the caudal and rostral aspects, matching the 9+ (*Kcng4*-) and 9− (*Kcng4* + ) subpopulations (Supplementary Video 1). The observed expression profile likely reflects the natural expression of the KCNG4 protein as the *Kcng4*^*Cre*^ line is a knock-in, and the patterned expression of *Kcng4* was previously suggested^38^. The *Kcng4*^*Cre*^ line also drives expression in a population of small Parvalbumin-positive (PV + ) neurons found in the lower half of the molecular layer (Fig. S3). Their distribution together with the TdTomato+ pinceau-like structures surrounding the axon initial segment of all PCs suggests that the *Kcng4*^*Cre*^ line also drives expression in basket cells^39^. We conclude that the *CaMKIIα*^*Cre/T29*^ and *Kcng4*^*Cre*^ lines allow us to label 9+ and 9− PC subpopulations of the flocculus, respectively.

### Distinct PC subpopulations are topographically connected with discrete brainstem pathways

**Fig. 3:**
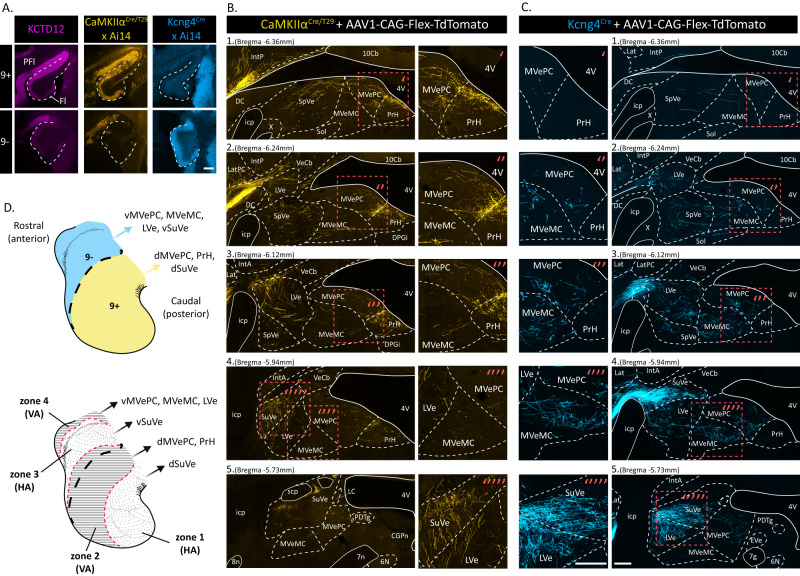
Anterograde Cre-dependent tracing from floccular regions 9+ and 9- reveals discrete long-range projections. **A** Transverse sections of the flocculus of *CaMKIIα*^*Cre/T29*^*;Ai14* (yellow) and *Kcng4*^*Cre*^*;Ai14* (blue) mice with KCTD12 (purple) immunolabeling. Scale bar = 200 µm. **B** and **C** TdTomato (Cre-dependent) labelled fibers in the vestibular structure following injection of AAV1-CAG-Flex-TdTomato in the flocculus of *CaMKIIα*^*Cre/T29*^ (yellow) or *Kcng4*^*Cre*^ (blue) animals. Scale bars = 200 µm. **D** Schematic of floccular domains based on PC molecular subpopulations (top) and functional zones described by Schonewille et al. (bottom). PrH prepositus hypoglossi, SuVe superior vestibular nucleus, LVe lateral vestibular nucleus, d/vMVePC dorsal/ventral medial vestibular parvicellular nucleus, MVeMC medial vestibular magnocellular nucleus, PFl paraflocculus, Fl flocculus, SpVe spinal vestibular nucleus, Lat lateral cerebellar nucleus, IntP nucleus interpositus posterior, IntA nucleus interpositus anterior, DPGi dorsal paragigantocellular nucleus of the reticular formation, Sol solitary nucleus, 4V fourth ventricle, Y group Y.

We next questioned how the two identified floccular PC subpopulations relate to the well-characterized functional zones associated with eye movements around both the horizontal and vertical axes, which project to separate downstream targets within the vestibular nuclei^28^. We co-injected a Cre recombinase-dependent reporter (AAV1-CAG-Flex-TdTomato) to label specific PC subpopulations, and a constitutive reporter (AAV5-CAG-ChR2-GFP) to verify the spread of the injection into both the *CaMKIIα*^*Cre/T29*^ and the *Kcng4*^*Cre*^ lines (Fig. S4). Floccular injections in *CaMKIIα*^*Cre/T29*^ mice resulted in TdTomato-labelled cells restricted to the 9+ subpopulation. These PCs densely project caudally to the VA-related nucleus prepositus hypoglossi (PrH) and the medial vestibular parvicellular nucleus (MVePC), primarily along the floor of the fourth ventricle and at the transition zone from PrH to MVePC, previously described as the “marginal zone”^40,41^, as well as to the HA-related dorsal superior vestibular nuclei (SuVe) (Fig. 3B). Labelled terminals in the medial vestibular magnocellular nucleus (MVeMC), spinal vestibular nucleus (SpVe) and lateral vestibular nucleus (LVe) were extremely scarce despite passing fibers. Injections performed in the *Kcng4*^*Cre*^ mice, resulted in TdTomato-labelled PCs restricted to the 9- subpopulation. Axon terminals were clearly observable in the MVeMC (VA), the ventral SuVe (HA) and dorsal LVe (VA). Some terminals could be found in the rostro-ventral MVePC, and were relatively scarce in the PrH (Fig. 3C). Therefore, 9+ PCs project primarily to the PrH, dorsal MVePC and dorsal SuVe, while the 9− PCs project largely to the MVeMC, ventral SuVe and LVe. Based on previous connectivity mapping^28^, the functional and molecular maps of the flocculus can be compared (Fig. 3D), indicating that these maps do not match. Thus, subpopulations of floccular PCs, identified based on KCTD12 expression, have clearly distinct downstream targets despite the relatively subtle differential AldoC expression.

The topographical distribution of mossy fiber (MF) terminals is known to relate to AldoC territories within the cerebellar vermis^42–44^. Whereas the topographical organization of the climbing fiber (CF) input to the functional zones of the flocculus is well-described and used to define VA and HA zones^28^, that of the MF terminals remains partly elusive^42–45^. To establish if MF input pathways relate to the 9+/9− subpopulations of the flocculus, we investigated a class of Somatostatin 28 immunoreactive (SST+) MFs, which is non-homogeneously distributed in the flocculus^46^. Serial immunostaining showed that in the flocculus, SST+ MF terminals predominantly populate the Hsp25+/KCTD12+ domain and expand in the Hsp25-/KCTD12+ domain (Fig. S5A). At the transition from 9+ to the 9− subpopulation, SST+ MFs were scarce, and became absent toward the rostral aspect of the flocculus. We found SST+ neurons in the PrH, MVePC, SpVe as well as the dorsal paragigantocellular reticular nucleus (DPGi) (Fig. S5B), all of which project to the flocculus. SST+ MFs are exclusively VGluT2+/VGluT1− (Fig. S5C), which is in accordance with the known expression of glutamate transporters in PrH, MvePC and SPVe neurons^47^, suggesting that SST+ MFs originate from either one or more of these nuclei. We next injected a Cre-dependent retrograde AAV (pAAV-CAG-FLEX-rc [Jaws-KGC-GFP-ER2]) unilaterally into the flocculus of an *SST*^*Cre*^ transgenic mouse line, to exclusively label the SST + MF terminals in the flocculus. We observed GFP-labelled MFs, distributed in a pattern identical to SST+ MFs observed through immunostaining, in both the injected flocculus and the contralateral non-injected flocculus (Fig. S5D). We identified GFP-labelled somata throughout the PrH and the dorsal aspect of MVePC, both ipsi- and contralaterally, with no other GFP-labelled cells observed in the SpVe, DPGi or any other brain region (Fig. S5D).

**Fig. 4:**
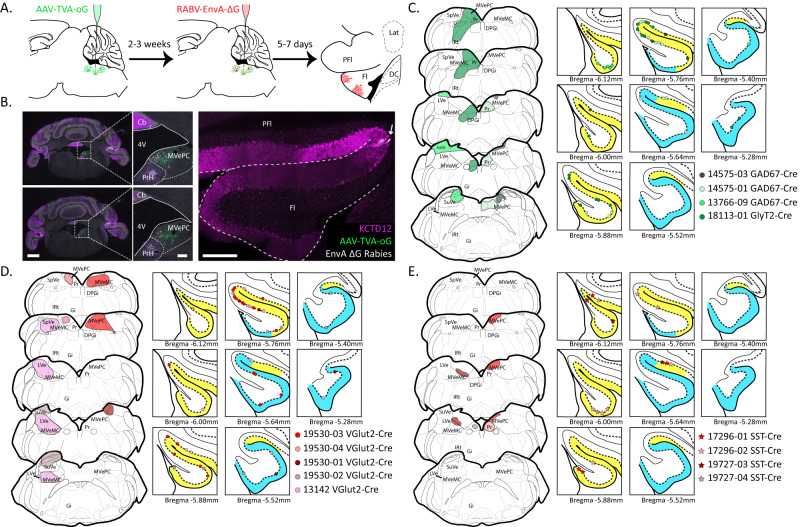
Monosynaptic rabies tracing from neural subpopulations in the vestibular complex and PrH. **A** Schematic of the procedure for monosynaptic tracing with genetically modified rabies virus. AAV helper (AAV1-EF1a-Flex-GTB or BA-96-AAV2/1-pAAV-Syn-Flex-nGToG-WPRE3) and rabies viruses (EnVA-G-deleted-Rabies-mCherry or BRABV-001-pSADB19dG-mCherry) were injected in the vestibular complex and/or PrH at different time points in either *GAD67*^*Cre*^, *GlyT2*^*Cre*^, *VGluT2*^*Cre*^ or *SST*^*Cre*^ mice. Retrograde trans-synaptically-labelled PCs are then identified in the flocculus. **B** Example of primary infected neurons in the PrH and MVePC (left, scale bar = 1 mm; middle, scale bar = 200 µm) following injections in a *SST*^*Cre*^ mouse, with trans-synaptically labelled 9 + PC in the flocculus (right, Scale bar = 200 µm). **C**–**E** Localization of the primary infected cells in *GAD67*^*Cre*^/*GlyT2*^*Cre*^ (**C**), *VGluT2*^*Cre*^ (**D**) or *SST*^*Cre*^ (**E**) mice. Each color represents a different injection, and associated marks in the flocculus represent single PCs identified following retrograde trans-synaptic labelling. Distribution of PC subpopulations in the flocculus are illustrated with yellow (9+) and blue (9−). PrH prepositus hypoglossi, SuVe superior vestibular nucleus, LVe lateral vestibular nucleus, MVePC medial vestibular parvicellular nucleus, MVeMC medial vestibular magnocellular nucleus, PFl paraflocculus, Fl flocculus, SpVe spinal vestibular nucleus, DPGi dorsal paragigantocellular nucleus of the reticular formation, Gi gigantocellular nucleus of the reticular formation, 4V fourth ventricle. Schematics adapted from Paxinos & Franklin, 2001^85^.

Previous studies suggested that the type of neurons targeted by floccular Purkinje cells in the vestibular structure, either glutamatergic, glycinergic or GABAergic neurons, are associated with segregated pathways of oculomotor control^40,48^. Therefore, we investigated whether 9+ and/or 9− PC sub-populations target neurons of a defined molecular identity in the vestibular nuclei. We injected genetically modified monosynaptic rabies^49^, subsequent to injection of helper AAVs (see Methods), within the vestibular nuclei of either *GAD67*^*Cre*^, *GlyT2*^*Cre*^, *VGluT2*^*Cre*^ or *SST*^*Cre*^ mice, and identified retrogradely labelled PCs in the flocculus (Fig. 4A, B). Primary infection of inhibitory (*GAD67*^*Cre*^ or *GlyT2*^*Cre*^) or excitatory (*VGluT2*^*Cre*^) floccular target neurons resulted in trans-synaptic labelling of both 9+ and 9− PCs in all cases (Fig. 4C, D). The distribution of labelled PCs throughout the flocculus for each injection is consistent with the location of primary infected cells as defined by our connectivity map (Fig. 3D). For example, injection 13766-09, in a *GAD67*^*Cre*^ animal, was located in the dorsal SuVe, with a minimal ventral spread. In this case, trans-synaptically labelled PCs were mainly observed in the most caudal aspect of 9+ (putative HA zone 1, see Fig. 3D), but cells could also be found in 9− (putative HA zone 3). Interestingly, all injections showed preferential labelling of PCs in the 9+ sub-population. This could result from differential efficiency of rabies trans-synaptic infection, differential toxicity of the rabies, or it could reflect an actual differentiation in connectivity, which would imply that 9+ subpopulation is more densely connected to the downstream targets. Nonetheless, these results reject the hypothesis that the 9+ or 9− subpopulation exclusively targets vestibular nuclei and/or PrH neurons of excitatory or inhibitory nature. Next, we genetically targeted the SST+ sub-population of excitatory neurons in the PrH/MVePC using *SST*^*Cre*^ mice (Fig. 4E). Selective primary infection of SST+ neurons resulted in trans-synaptic labelling of only 9+ PCs. Each injection showed sparse PC trans-synaptic labelling, but no trans-synaptic labelling of 9- PCs was observed (9+ PCs: 16 vs. 9− PCs: 0, in 4 mice). As a comparison, even a small injection in a similar region of a *VGluT2*^*Cre*^ mouse like in 19530-04 (Fig. 4D) resulted in sparse trans-synaptic PC labelling distributed between 9+ and 9− subpopulations. The relatively low number of labelled PCs could be due to the efficiency of primary infection for such a small population of neurons or due to its low connectivity. These data show that both afferent and efferent connections to 9+ and 9- subpopulations are topographically organized in segregated pathways.

### Local networks of floccular regions

We next investigated the local cytoarchitecture of the flocculus. PLCβ4 immunostaining reveals subpopulations of PCs but also labels UBCs^50^, a group of excitatory interneurons densely populating the granular cell layer (Figs. S2C, S6A). UBC subpopulations express either PLCβ4, Calretinin (Calrt) or mGluR1α. We observed that PLCβ4+ and Calrt+ UBCs both predominantly populate the 9+ and are sparser in the 9− PC domain (Fig. S6A,B). Immunostaining for Tbr2, a putative marker for all UBCs^51^, visualizes this distribution (Supplementary Video 2). The confinement of UBCs along the flocculus matches the one observed in lobule IX where the UBC population is dense in AldoC-positive domains but relatively scarce in negative domain (Fig. S6C). Next, we investigated the distribution of another population of neurons unique to the flocculus, the Basal Interstitial Nucleus of the cerebellum^52^. We did not identify a specific distribution mirroring the distribution of 9+ or 9− PC subpopulations for these neurons that are nested in the white matter (Supplementary Video 2), suggesting that certain pathways remain common to both sub-populations.

PCs provide direct inhibitory feedback within the cerebellar cortex through collaterals onto other PCs, MLI, or UBCs. In the vermis, these collaterals were shown to stretch over several folia and reach distant regions hundreds micrometer away while remaining restricted to narrow parasagittal bands and therefore likely contained within a single AldoC band^53^. Possible transverse connections have been described between hemispheric and paravermal regions suggesting that collaterals can cross over cerebellar modules boundaries^54,55^. Injection of AAV1-CAG-Flex-TdTomato in *CaMKIIα*^*Cre/T29*^ mice reveals PC collaterals and demonstrates that 9+ PCs cross the boundary to contact 9− PCs (Fig. S7) and putatively other cell types within the granular cell layer. They branch profusely at the level of the PC layer in what Cajal originally described as the “infraganglionic plexus”^56^, the network of PC collaterals just below the PC layer. Similar tracing analysis in *Kcng4*^*Cre*^ was not possible, as molecular layer interneurons are also labeled and represent a dense population of inhibitory synapses in the infraganglionic plexus. This demonstrates a cross connectivity between PC subpopulations, and therefore across cerebellar modules directly at the level of the cortex.

### PC subpopulations mediate distinct kinematic properties

Next, to study the functional contribution of these distinct microzones, we optogenetically stimulated PC subpopulations of the flocculus and recorded the induced vertical and horizontal ipsilateral eye position. Activation of channelrhodopsin-expressing PCs has been demonstrated to drive various movements^29,30,32,57–59^. We used *Kcng4*^*Cre*^ and *CaMKIIα*^*Cre/T29*^ mice crossed with the transgenic Ai27 line to drive the expression of channelrhodopsin 2 (ChR2) in either the 9− or 9+ region of the flocculus, respectively. *Pcp2*^*Cre*^*;Ai27* mice were used to express ChR2 in all PCs and compare the activation of the whole flocculus with the activation of restricted domains. In the presence of Cre, the expression of ChR2 is consistently robust^29,30,32,57–59^. However, given the expression in putative basket cells in the *Kcng4*^*Cre*^ line, we first set out to determine the consequence of optogenetic stimulation in this line. We hypothesized, based on the expression of *Kcng4* (Fig. S3A–C), that optogenetic stimulation of the *Kcng4*^*Cre*^;*Ai27* flocculus would excite PCs in the 9− domain, while simultaneously exciting putative basket cells leading to the inhibition of the 9+ domain. We first confirmed this hypothesis ex vivo by recording PCs in floccular slices using a 5-ms LED pulse (Fig. S8A–C). This optogenetic stimulation excited Kcng4+ PCs while inhibiting Kcng4- PCs (Fig. S8A–C), presumably due to excitation of the basket cells that inhibit PCs. Analysis of the input-output curve based on current injection steps revealed significantly higher excitability in Kcng4+ PCs compared to Kcng4- PCs (*p* < 0.0001, Fig. S8D, E), similar to AldoC- compared to AldoC+ PCs (see Fig. 1H). In vivo, optogenetic stimulation identified two types of PC responses, with an increase and a suppression in SS frequency in *Kcng4* + PCs and *Kcng4*- PCs, respectively (Fig. S8F, G). Juxtacellular biocytin injections confirmed the nature of the recorded cells in histological post-processing. In comparison, in *Pcp2*^*Cre*^*;Ai27* animals, all PCs exhibited an increase of SS frequency during optogenetic stimulation (Fig. S8G). Optogenetic stimulation corresponds with an approximately 3-fold increase in SS firing frequency for all excited PCs found either in the *Kcng4*^*Cre*^;*Ai27* or *Pcp2*^*Cre*^*;Ai27* mice, and a 75% decrease for inhibited PCs found only in *Kcng4*^*Cre*^;Ai27 mice. The similar results observed for excited PCs in *Kcng4*^*Cre*^;*Ai27* and *Pcp2*^*Cre*^*;Ai27* mice suggest that in *Kcng4*^*Cre*^;*Ai27* mice the activation of interneurons has little to no impact on the *Kcng4* + PCs during the optogenetic stimulation; any inhibition appears to be overruled by the strong direct excitation. We conclude that illumination of the flocculus in *Kcng4*^*Cre*^*;Ai27* mice will lead to simultaneous activation of 9− and inhibition of 9+ PCs (Figs. S3 and S8 and Methods), while illumination of the flocculus in the *CaMKIIα*^*Cre/T29*^*;Ai27* mice will result only in the excitation of 9+ PCs (Figs. 2 and S9).

**Fig. 5:**
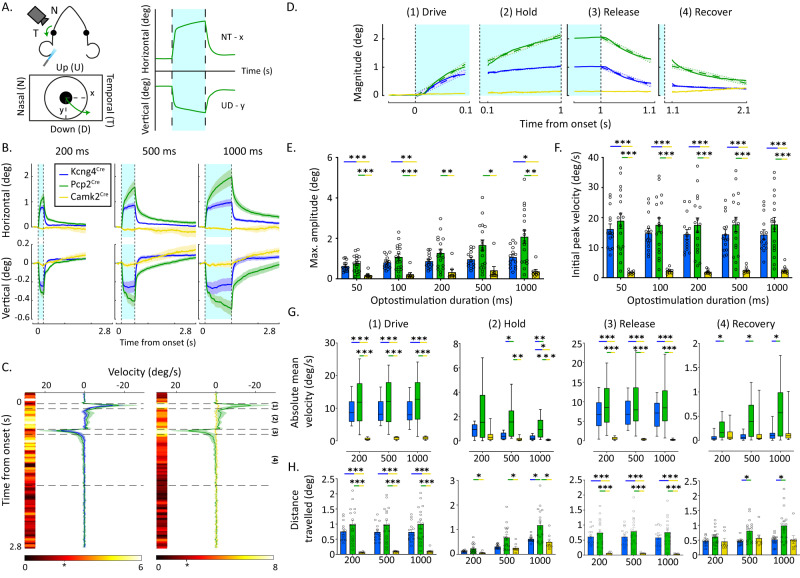
Selective optogenetic stimulation of the 9+ and 9− PC subpopulations drives distinct kinematics. **A** Schematic image of the recording settings. The left flocculus is optogenetically stimulated (blue shading indicates stimulation time) while simultaneously recording the ipsilateral eye position in X and Y coordinates. The green arrows indicate the direction of the eye movement during optogenetic stimulation of all PCs in *Pcp2*^*Cre*^*;Ai27* animals. The horizontal eye movement (naso-temporal, in degrees) is shown as a positive deflection. The vertical eye movement (downward) is shown as a negative deflection. **B** Means of horizontal (top) and vertical (bottom) eye movement traces for *Pcp2*^*Cre*^*;Ai27* (green), *Kcng4*^*Cre*^*;Ai27* (blue), and *CaMKIIα*^*Cre/T29*^*;Ai27* (yellow) mice. Optogenetic stimulation durations are 200, 500 or 1000 ms, indicated by blue shading. **C** Velocity profiles of mean magnitude eye movement response during 1000-ms optogenetic stimulation. The velocity profile shows a peak after LED onset and LED offset. The heatmap shows the statistical divergence in velocity at each time point using the *t*-score from the unpaired two-tailed Student’s *t* test. Velocity is statistically significant when the *t*-score surpassed t > 2.043 for *Pcp2*^*Cre*^*;Ai27* / *Kcng4*^*Cre*^*;Ai27* (df = 30), and t > 2.064 for *Pcp2*^*Cre*^*;Ai27* / *CaMKIIα*^*Cre/T29*^*;Ai27* (two-tailed *t*-value), indicated by the asterisks. Dotted lines indicate movement phases based on peak velocity. **D** Representation of the four phases (1) drive, (2) hold, (3) release, and (4) recovery obtained from the velocity traces, displaying the absolute magnitude of the movement for optogenetic stimulation of 1000 ms. Blue shading indicates the time period of optogenetic stimulation. **E** Maximum amplitude of the eye movement response reached at the end of optogenetic stimulation for all optogenetic stimulation durations. **F** Initial peak velocity of the magnitude eye movement response occurring shortly after LED onset. **G** Absolute mean velocity in the four phases of the eye movement. **H** Distance traveled by the eye during the four phases of the induced eye movement. ***: *p* < 0.001, **: *p* < 0.01; *: *p* < 0.05 05 based on two-way ANOVA with multiple comparisons (see Supplementary Table 1). *n* = 14 *Kcng4*^*Cre*^*;Ai27* mice, 15 *Pcp2*^*Cre*^*;Ai27* mice and 8 *CaMKIIα*^*Cre/T29*^*;Ai27* mice. All data are presented as mean values ± SEM except (**G**), which is presented as box plots where the median is shown as the line, boxes extend from 25th to 75th percentile and whiskers extend to min and max values.

Next, we activated floccular PCs in vivo by illuminating the 9+/9− microzones with blue (470 nm) light through a surgically prepared opening in the petrosal bone (Fig. S10). Activation of PCs in the flocculus reliably induced diagonal eye movements in *Kcng4*^*Cre*^*;Ai27*, *CaMKIIα*^*Cre/T29*^*;Ai27* and *Pcp2*^*Cre*^*;Ai27* mice (Fig. 5A). The maximum amplitude of the horizontal movement was larger than that of the vertical eye movement in all genotypes and this difference was comparable among genotypes (Fig. 5B). To reduce the dimensionality of the eye movement and attain a single measure for eye position, we calculated the magnitude position of the eye. Direct comparison of the maximum amplitude and initial peak velocity revealed that *CaMKIIα*^*Cre/T29*^*;Ai27* mice make smaller and slower eye movements than *Kcng4*^*Cre*^*;Ai27* and *Pcp2*^*Cre*^*;Ai27* mice (Fig. 5B, D and E). Subsequent analyses revealed that the kinematics of the movements were significantly different between genotypes. To quantify this, we divided longer optogenetically-elicited eye movements (≥200 ms) into four components based on peaks in movement velocity: drive, hold, release, and recovery (Fig. 5C and F). The drive and release phases are at the onset and offset of the optogenetic stimulation, time-locked with the highest velocity eye movements and last ~100 ms (Fig. 5F). The high velocity drive phase is followed by a relatively low velocity hold phase during the period of the optogenetic stimulation. The release phase is the relatively high velocity period after the offset of the optogenetic stimulation and the recovery phase occurs as the eye returns to its original position. During the high velocity drive and release phases, we found no differences in absolute velocity between *Kcng4*^*Cre*^*;Ai27* and *Pcp2*^*Cre*^*;Ai27* mice, suggesting the involvement of 9− PCs in fast movements (Fig. 5G). However, the two phases with lower velocities, hold and recovery, both exhibited significantly different kinematics. Conversely, driving the 9+ PCs in *CaMKIIα*^*Cre/T29*^*;Ai27* mice resulted in slower movements (Fig. 5G). These findings were confirmed when we compared the distance traveled during each of the phases (Fig. 5H). Stimulation in *Kcng4*^*Cre*^*;Ai27* mice resulted in larger distances traveled than those evoked by 9+ PCs in the *CaMKIIα*^*Cre/T29*^*;Ai27* mice, but only during the high velocity drive and release phases, suggesting that the latter 9+ PCs are involved in slow, sustained movements. The larger movement observed in *Kcng4*^*Cre*^*;Ai27* mice may reflect the activation of a higher number of PCs. To test this hypothesis, we have performed stepwise stimulation by lowering down light intensity onto *Kcng4*^*Cre*^*;Ai27* mice (Fig. S10D,E). Even at only 20% of the original power, *Kcng4*^*Cre*^*;Ai27* mice conserve the fast kinetic component, while the overall amplitude is reduced, rejecting the hypothesis of a population size effect. Thus, elicited eye movements exhibit both fast and slow movement components that can be differentially driven by distinct PC subpopulations.

Optogenetic manipulation in *Kcng4*^*Cre*^*;Ai27* mice drives direct activation of 9- PCs together with indirect inhibition of 9+ PCs (Fig. S8). To test the effect of floccular PC inhibition on eye movements, we used *CaMKIIα*^*Cre/T29*^*;Ai39*, *Kcng4*^*Cre*^*;Ai39* and *Pcp2*^*Cre*^*;Ai39* mice, in which the Cre-dependent expression of the inhibitory opsin Halorhodopsin (Ai39^60,61^) allows for light-induced hyperpolarization of neurons (Fig. S11). Direct inhibition of PCs in *CaMKIIα*^*Cre/T29*^*;Ai39, Kcng4*^*Cre*^*;Ai39* and *Pcp2*^*Cre*^*;Ai39* mice had no detectable effect on the pupil position, arguing against a prominent upstream effect of basket cell activation, therefore 9+ PCs inhibition, in the *Kcng4*^*Cre*^*;Ai27* mice.

**Fig. 6:**
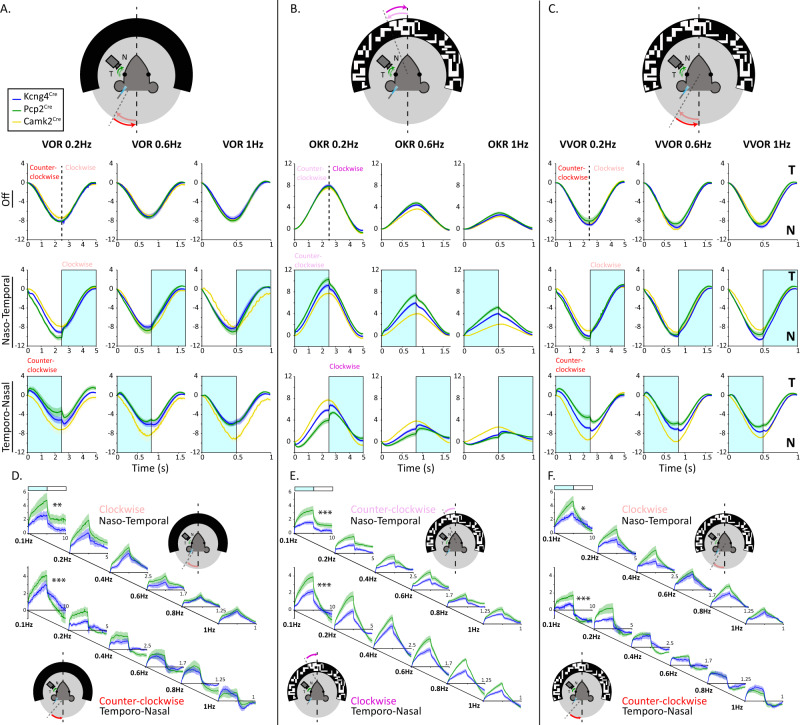
Selective optogenetic stimulation of the 9+ and 9− PC subpopulations differentially influences sensory input-driven eye movements. **A**–**C** Schematic of the VOR, OKR and VVOR experimental design (top). During sensory stimulation in *Pcp2*^*Cre*^*;Ai27* (green), *Kcng4*^*Cre*^*;Ai27* (blue), and *CaMKIIα*^*Cre/T29*^*;Ai27* (yellow) mice, optogenetic stimulation was either absent or provided during the portion in which sensory stimulation evoked a naso-temporal (light red, light purple) or temporo-nasal (dark red, dark purple) eye movement. Optogenetic activation of (sub)populations of PCs during vestibular stimulation (VOR), visual stimulation (OKR), or both (VVOR) drove differential responses between genotypes. Temporal (T) and the Nasal (N) side for the direction of the eye are indicated on the right. **D**–**F** Subtraction of eye movements during sensory stimuli in the absence of optogenetic stimulation from those with optogenetic stimulation to compare the impact of stimulation in *Pcp2*^*Cre*^*;Ai27* (green) and *Kcng4*^*Cre*^*;Ai27* (blue) mice. ***: *p* < 0.001, **: *p* < 0.01; *: *p* < 0.05 based on two-way ANOVA with multiple comparisons (see Supplementary Table 1). *n* = 10 *Kcng4*^*Cre*^*;Ai27* mice, 15 *Pcp2*^*Cre*^*;Ai27* mice and 8 *CaMKIIα*^*Cre/T29*^*;Ai27* mice. All data are presented as mean values ± SEM.

The primary function of PCs is to modulate a motor response generated by other brain regions. Therefore, we tested the effect of optogenetic activation during sensory evoked eye movement (Fig. 6). We measured the eye movement evoked by oscillating vestibular (to evoke a vestibulo-ocular reflex, VOR), visual (to evoke an optokinetic reflex, OKR) or visuo-vestibular stimulation (to evoke a visual vestibulo-ocular reflex, VVOR) across a range of frequencies (0.1–1.0 Hz), coupled or not with optogenetic stimulation. Without optogenetic stimulation (“Off”) all genotypes presented similar responses to all sensory stimulations, at all frequencies (Fig. 6A–C). Optogenetic activation during clockwise OKR and counterclockwise VOR and VVOR induces naso-temporal eye movements that are enhanced by light stimulation in both *Pcp2*^*Cre*^*;Ai27* and *Kcng4*^*Cre*^*;Ai27* mice. No measurable effects of optogenetic activation were observed in *CaMKIIα*^*Cre/T29*^*;Ai27* mice under any of the conditions. Interestingly, the effect of optogenetic activation in *Pcp2*^*Cre*^*;Ai27* and *Kcng4*^*Cre*^*;Ai27* mice consists of an initial fast naso-temporal movement, followed by a slower, more gradual drift. Overall, the responses of *Pcp2*^*Cre*^*;Ai27* and *Kcng4*^*Cre*^*;Ai27* mice appear similar yet of different magnitudes, but this difference in magnitude is frequency dependent with a significant difference only apparent during the slowest behavioral stimulation (Fig. 6). Subtracting the eye movements evoked in the absence of light stimulation (Fig. 6D–F) reveals that for VOR, OKR and VVOR the relative difference between *Pcp2*^*Cre*^*;Ai27* and *Kcng4*^*Cre*^*;Ai27* mice was larger for slower sensory evoked eye movements. These data demonstrate that, similar to stimulation at rest, the population of cells recruited in *Kcng4*^*Cre*^*;Ai27* mice during short-lasting stimulation while providing high frequency sensory input is sufficient to reproduce the effect of the optogenetic stimulation of all PCs in *Pcp2*^*Cre*^*;Ai27* mice.

## Discussion

In this study, we show that minor nuances in neural molecular identity reflect physiological, anatomical and functional distinctions. In the cerebellum, the specific functional contribution of PC subpopulations has remained mostly unresolved making the role of the parallel microzones that they form unclear. Do the microzones control distinct behaviors or act in concert for the execution of a motor task? If operating together, do they control antagonist muscle sets or different components of the movement? To answer these questions, we examined regions with different gene expression levels in the flocculus and identified intrinsic physiological differences between PC subpopulations, different projection pathways into and out of the flocculus as well as different kinematic features of the movements in the same direction upon optogenetic stimulation.

The AldoC protein is currently the main molecular marker of reference to study PC subpopulations. However, the compartmentalization of PCs has been continuously studied and redrawn^10,11^. The high contrast of KCTD12 expression confirms that the nuances of AldoC expression are relevant in that electrophysiological features indicate the 9− domain is not a conventional AldoC- domain. Similar properties can be inferred in other domains where AldoC expression nuances suggest more diversity in PC intrinsic properties, such as in the b + /3 + /3b + /f + /e1 + /e1− and e2+ domains^11^. In the flocculus, we observed two non-overlapping PC subpopulations that are either AldoC+/KCTD12+/Hsp25+ or AldoC+/KCTD12+/Hsp25−, both PLCβ4-/Nfh- and together forming the 9+ subpopulation, and a third subpopulation that is PLCβ4+/Nfh+ forming the 9- subpopulation. In the nodulus, the differential patterning of these markers describes a fourth population that is AldoC+/Nfh+/Hsp25-^12^. The presence of these complex variations in molecular footprint indicates a high diversity of PCs, which has been confirmed with novel single cell RNA sequencing^38^. Our data show that AldoC expression and electrophysiological properties of PCs both exhibit correlated continuums of variability, properties in which membrane current regulators including KCTD12 or KCNG4 may also play an important role. Therefore, the cerebellar cortex may be described as a genetically multimodal structure linked to spiking activity outcomes. Such a continuum in protein levels and electrophysiological properties has also been observed in interneurons of cerebral cortex^62^, striatum^63^ and hippocampus^64^. However, divergent long-range projections among such continuums of neuronal subpopulations have not been highlighted before. Indeed, despite the graded physiological changes between 9+ and 9−, we observe a relatively clear compartmentalization of afferent and efferent projections. This suggests that Purkinje cells are indeed molecularly and electrophysiologically tuned to their inputs and outputs in order to properly contribute to motor function. Novel approaches combining molecular features and high-resolution anatomical connectivity^65^ should be used in parallel with physiological studies to understand the functional relevance of diversity in molecular signatures.

Several lines of evidences led us to hypothesize that specific behaviors are mediated by an ensemble of parallel cerebellar microzones of different molecular identities. Trans-synaptic retrograde tracing from muscles to the cerebellum has shown that AldoC+ and Aldoc− compartments converge onto individual muscles^20^. Moreover, neuronal mapping of the vestibulo-ocular system of the pigeon cerebellum revealed that each individual functional zone in the flocculus and the uvula encompass both an AldoC+ and an AldoC− PC domain^21^. Based on the distribution of PC subpopulations relative to known functional zones (Fig. 2D), similar conclusions can be reached in mice where HA and VA functional units consist of both 9+ and 9− PCs^28^. Therefore, all directions of compensatory eye movements are likely to involve multiple PC subpopulations. The anatomical connectivity we reveal supports this model. We show that a group of 9+ PCs, putatively belonging to VA zone 2^28^, projects to the PrH and MVePC, also known as the marginal zone. This zone has been described to send excitatory projections to the ipsilateral oculomotor nucleus, which in turn controls the ipsilateral medial rectus muscle^40,66^. In parallel, a group of the 9- PCs, putative part of VA zone 4, projects to the MVeMC known to send inhibitory projections to the abducens nucleus, which controls the ipsilateral lateral rectus muscles. This supports the concept that the two muscles required for the horizontal eye movement are modulated by (parts of) the 9+ and 9− domains^48^. Given this circuit configuration, ipsilateral 9+ PCs would inhibit glutamatergic MVePC/PrH neurons leading to relaxation of the ipsilateral medial rectus, while 9− PCs inhibit glycinergic neurons in the MVeMC, leading to disinhibition, and thus contraction, of the ipsilateral lateral rectus. Our optogenetic data support this hypothesis as optogenetic stimulation of 9+ PCs leads to a slow drifting movement, possibly from medial rectus relaxation, while the optogenetic stimulation of 9− PCs leads to a fast naso-temporal movement, likely due to lateral rectus contraction. The additional slower movement observed when both populations are stimulated compared to only 9− PCs would in that case be due to the fact that the medial rectus muscle is not (completely) relaxed. However, more complex functional relationships through other pathways are conceivable in view of the heterogeneity of the vestibular nuclei neurons, their inter-commissural projections, and the intertwined projections to the oculomotor and abducens nuclei. Previous rabies tracing from the rectus muscles in guinea pigs and rats indicated that zone 2 (9 + ) and 4 (9−) both may control lateral and medial rectus muscles through circuits with a distinct number of synaptic steps^67,68^. The connection from the abducens nucleus to the contralateral oculomotor nucleus supports the idea that zone 4 (9−) controls not only the ipsilateral lateral rectus but also the contralateral medial rectus. Similar conclusions can be drawn for vertical eye movements, for which 9+ and 9− PCs, putatively belonging to HA zone 1 and 3, project to the dSuVe and vSuVe, respectively. These subnuclei in turn control the superior and inferior rectus muscles^66^ through the oculomotor nucleus, suggesting a possible general design of the neuronal pathway from these zones to the rectus muscles. The afferent pathways to 9+ and 9− PCs may also give insight into the type of information they receive and convey. The density of UBCs in the 9+ zone points toward a predominantly vestibular input. In contrast, the nucleus reticularis tegmenti pontis (NRTP), known to transmit visual information, was found to preferentially project to the rostral half of flocculus in cats that, assuming a similar configuration, would align with the 9− microzone in the mouse^45^. So, while 9+ and 9− domains remain relatively compartmentalized, it should be noted that we also found evidence for transversal connectivity through PC collaterals, offering routes for mutual interactions.

Previous studies demonstrate that optogenetic stimulation in the cerebellum induces or alters movement kinematics^29,30,32,57–59^. PCs are known to encode kinematic features of movement such as speed and direction^69–73^. In fact, the cerebellum not only encodes kinematic features of movement but also directly contributes to those kinematics^24^. Although the modular organization of the cerebellum has been associated with distinct motor behaviors^18^, understanding of the relevance of that organization for the production of multi-component movements is limited. Thus far, optogenetic strategies have not had the refined spatial resolution to exclusively modulate activity in PC microzones. We find that the optogenetic activation of 9− subpopulation is capable of driving fast eye movements, whereas the movement induced by activation of the 9+ subpopulation is much smaller and slower. The similarity of response between *Pcp2*^*Cre*^*;Ai27* and *Kcng4*^*Cre*^*;Ai27* mice in the fast components of the movement (“Drive” and “Release”), together with the similar response to optogenetic activation during high frequency sensory-evoked eye movements, suggests that 9− PCs have a predominant role in faster components of the behavioral response. The minimal movement following activation in *CaMKIIα*^*Cre/*T29^ mice suggests that the 9+ subpopulation may require prior or concomitant activation of 9− subpopulation to modulate the slow components of the movements. In addition to differential control of opposing muscles, this finding could also indicate a driver-modulator differentiation observed in e.g., corticothalamic projections^74^, but can also be linked, for example, to the phasic-tonic characteristic of the pulse-step signal of the oculomotor nuclei that drive eye movements^75^. This remains to be resolved. We hypothesize that while the ipsilateral flocculus stimulation drives naso-temporal movements through 9− PCs and modulates it via 9+ PCs, the contralateral activation may do the same for temporo-nasal movement where 9− PCs would act on the fast drive of the medial rectus and 9+ on the slow relaxation of the lateral rectus, as supported by the connectivity map to the ipsi- and contralateral structures described above. Our data demonstrate that PC subpopulations can act in parallel to precisely drive muscle activity for specific movement. Given the broad range of behaviors that involve the cerebellum, it is likely that the individual contributions of discrete subpopulations will vary across different microzones. While optogenetic stimulation within the flocculus at rest leads to a downward and naso-temporal movement time-locked to the onset of PC excitation^29,30,32^, within the cerebellar vermis and paravermis, suppression of PC firing leads to graded disinhibition of CN neurons and a movement time-locked to the stimulus onset, and PC activation leads to movement time-locked to stimulus offset, when rebound firing of the PCs occurs^57,58^.

All movements are a composite of multiple kinematic components, which can be encoded by different neurons within the basal ganglia^2^ and motor cortex^3^. In our study, we dissect the movement kinematics in more detail and show that cerebellar PC subpopulations that are part of the olivocerebellar modules drive different components of movement. We provide empirical evidence that intrinsically different microzones in the cerebellum are designed to contribute to the whole of a multiplexed movement. While the classification of neuronal subpopulations rapidly expands, our data demonstrate the behavioral relevance of discrete clusters of neurons.

## Methods

### Animals

All animals in this study were handled and kept under conditions that respected the guidelines of the Dutch Ethical Committee for animal experiments and were in accordance with the Institutional Animal Care and Use Committee of Erasmus MC (IACUC Erasmus MC), the European and the Dutch National Legislation. All animals were maintained under standard, temperature controlled, laboratory conditions. Male and female, adult mice were included at random in the study. Mice were kept on a 12:12 light/dark cycle and received water and food ad libitum. B6.Cg-Tg(Pcp2-cre)3555Jdhu/J (*Pcp2*^*Cre*^, Stock #010536)^76^, B6.Cg-Tg(Camk2a-cre)T29-1Stl/J (*CaMKIIα*^*Cre/T29*^, Stock #005359)^77^, and B6.129(SJL)-Kcng4tm1.1(cre)Jrs/J (*Kcng4*^*Cre*^, Stock #029414)^78^, Sst^tm2.1(cre)Zjh^/J (*SST*^*Cre*^, Stock #013044), B6.Cg-Gt(ROSA)26Sort^m27.1(CAG-COP4*H134R/tdTomato)Hze^/J (Ai27, Stock #012567)^79^, B6.Cg-Gt(ROSA)26Sort^m14(CAG-tdTomato)Hze^/J (Ai14, Stock #007914)^80^ were purchased from the Jackson Laboratory. All mouse lines were maintained and used with heterozygous expression of transgenic alleles. Mouse lines were crossed to obtain *Pcp2*^*Cre*^*;Ai27*, *Kcng4*^*Cre*^*;Ai27*, *CaMKIIα*^*Cre/T29*^*;Ai27*, *Pcp2*^*Cre*^*;Ai39*, *Kcng4*^*Cre*^*;Ai39*, *CaMKIIα*^*Cre/T29*^*;Ai39*, *Kcng4*^*Cre*^*;Ai14*, *CaMKIIα*^*Cre/T29*^*;Ai14* mice described in this work.

### Immunohistochemistry and histology

For all immunohistochemical and histological experiments, except filled cells from ex vivo recordings (see below), animals were deeply anesthetized through intraperitoneal administration of sodium pentobarbital (60 mg/ml), directly followed by transcardial perfusion with saline and then 4% paraformaldehyde (PFA) in 0.12 M phosphate buffer (PB), pH = 7.6. Brains were post-fixed for 1 h in 4% PFA at room temperature (RT), transferred to a 10% sucrose/ PB solution overnight at 4 °C. The brains were embedded in a 10% gelatin (FUJIFILM Wako Pure Chemicals)/10% sucrose mix, gelatin blocks were incubated in 30% sucrose/10% formaldehyde for 2 h at RT and incubated overnight in 30% sucrose at 4 °C. Subsequently, 40 μm-thick coronal or sagittal sections were cut with a freezing microtome. Free-floating sections were rinsed with 0.1 M PB and incubated in 10 mM sodium citrate (pH 6) at 80 °C for 2 h, for antigen retrieval. Sections were rinsed with 0.1 M PB, followed by 3 washes of 10 min in phosphate-buffered saline (PBS). Then, sections were incubated for 90 min at RT in a solution of PBS/0.5%Triton-X100/10% normal horse serum to block nonspecific protein-binding sites, and incubated 48 h at 4 °C in a solution of PBS/0.4% Triton-X100/2% normal horse serum (NHS), with primary antibodies diluted as follows: KCTD12^34,81^ 1:500 (rabbit polyclonal, 15523-1-AP, ProteinTech), KCTD12^34,81^ 1:200 (mouse monoclonal, sc-271855, SantaCruz Biotech.), AldolaseC^82^ 1:500 (goat polyclonal, SC-12065, Santa Cruz Biotech.), Hsp25^83^ 1:1000 (rabbit polyclonal, SPA-801, Stressgen), GFP 1:1000 (chicken polyclonal, GFP-1020, Aves), RFP 1:1000 (guinea pig polyclonal, 390005, Synaptic Systems), Parvalbumin 1:7000 (rabbit polyclonal, PV-25, Swant), SST (chicken polyclonal, 366006, Synaptic Systems; and goat polyclonal, sc-7819, SantaCruz), Calretinin (mouse monoclonal, Swan 6B3-GEL), Tbr2 1:1000 (chicken polyclonal, AB15894, MiliP). After rinsing in PBS, sections were incubated for 2 h at RT in PBS/0.4% Triton-X100/2% NHS solution with secondary antibodies coupled with Alexa488, Cy3 or Cy5 (1:200, Jackson ImmunoResearch). Sections were mounted on coverslip in a solution of gelatin/chrome alum and cover-slipped with Mowiol (Polysciences Inc.). To identify recorded cells from ex vivo recordings, slices were transferred after recording/filling PCs to 4% paraformaldehyde for at least 24 h followed by successive washes of 0.1 M PB before going through the standard immunohistological protocol. To identify recorded sites from in vivo recordings, free-floating sections were thoroughly rinsed with 0.1 M PBS (5 × 10 min, pH 7.6) and immediately incubated in a 0.1 M PBS/2% NHS/0.4% Triton-X100 solution with secondary antibodies coupled with streptavidin-Cy3 (1:200, Jackson ImmunoResearch) for 2 h at RT. Next, the sections were rinsed, stained with DAPI, and mounted as described above.

Brains were cleared using an adapted iDISCO protocol^84^. Brains were washed in PBS (1.5 h), followed by successive methanol/H_2_O steps of 20%-50%-80%-100%-100%, 1 h each. Samples were treated with a solution of dichloromethane (DCM) and 100% methanol (2:1) for 1 h. Brains were then bleached with 5% H_2_O_2_ in 90% methanol (ice cold) at 4 °C overnight. After bleaching, samples were successively washed in 80%-50% methanol/H_2_O, then 40%-20% methanol/PBS for 1 hr each, and finally in PBS/0.2% Triton X-100 for 1 h two times. After rehydration, samples were pre-treated in a solution of PBS/0.2% Triton X-100/20% DMSO/0.3 M glycine at 37 °C for 36 h, then blocked in a mixture of PBS/0.2% Triton X-100/10% DMSO/6% donkey serum at 37 °C for 48 h. Brains were incubated in primary antibody in PTwH solution (PBS/0.2% Tween-20/5% DMSO/3% donkey serum with 10 mg/ml heparin) for 7 days at 37 °C with primary antibody: SST (goat polyclonal, sc-7819, SantaCruz), NECAB1 1:1000 (rabbit polyclonal, HPA023629, Atlas Antibodies), and Tbr2 1:1000 (chicken polyclonal, AB15894, MiliP) together, or RFP (rabbit polyclonal, 600-401-379, Rockland) alone. Amphotericin was added once every 2 days at 1 µg/ml to avoid bacterial growth. Samples were then washed in PTwH six times (1 h each, after the fourth wash leave it at room temperature overnight, then two washes on the following day) followed by the second round of incubation with primary antibody for 7 days. Brains were then washed in PTwH, 6 washes in 24 h, as described before, then incubated in secondary antibody in PTwH/3% donkey serum at 37 °C for 7 days with secondary antibodies at 1:750. Brains were then washed in PTwH, 6 washes in 24 h, again, followed by successive washes in methanol/H_2_O steps of 20%-40%-60%-80%-100%-100% for 1 h each, and finally incubation overnight in a solution of DCM and100% methanol. For tissue clearing, brains were incubated 20 mins in DCM, twice, and conserved in Benzyl ether at room temperature.

### Imaging acquisition and analysis

Images were acquired with an upright Imager.M2 (Zeiss) equipped with a 10× and 20× lenses, for fluorescent microscopy, and a confocal microscope LSM700 (Zeiss) with upright 20×, 40× and 63× lenses. Images were treated with a Photoshop routine for overall brightness and signal level, all treatment being equally applied on the entire images. For serial reconstruction, we performed an identical immunostaining process (fixation, incubation time), identical image acquisition (gain, exposure time, resolution), and identical image adjustment for analysis (brightness and contrast values) per marker, all performed in parallel in a single session for all markers tested. iDISCO samples were imaged in horizontal orientation with an UltraMicroscope II (LaVision BioTec) light sheet microscope equipped with Imspector (version 5.0285.0) software (LaVision BioTec). Images were taken with a Neo sCMOS camera (Andor) (2560 × 2160 pixels. Pixel size: 6.5 × 6.5 μm2). Samples were scanned with double-sided illumination, a sheet NA of 0.148348 (resuls in a 5 μm thick sheet) and a step-size of 2.5 μm using the horizontal focusing light sheet scanning method and using the contrast blending algorithm. Following laser filter combinations were used: Coherent OBIS 488–50 LX Laser with 525/50 nm filter, Coherent OBIS 561–100 LS Laser with 615/40 filter, Coherent OBIS 647–120 LX with 676/29 filter.

### Viral injections

For stereotaxic injections of Adeno-Associated Virus (AAVs) in the mouse flocculus, animals (*CaMKIIα*^*Cre/T29*^: *n* = 3; *Kcng4*^*Cre*^: *n* = 4) were anesthetized with a mixture of isoflurane/oxygen (5% for induction, 1.5–2.0% for maintenance), carprofen (Rimadyl Cattle i.p. 5 mg/kg), buprenorphine (Temgesic, i.p. 0.05 mg/kg), lidocaine (s.c. 0.4 mg/ml) and bupivacaïne (s.c. 0.1 mg/ml) were applied to reduce perisurgical pain and inflammation. Ophthalmic ointment was applied to the eyes to prevent corneal drying and damage. Body temperature was monitored and kept constant at 37 °C throughout the entire surgical procedure. Mice were positioned on a stereotaxic frame with a mouse gas anesthesia head holder (David Kopf Instruments, model 933-B). After opening of the scalp, a craniotomy (diameter ~1.5 mm) was made onto the interparietal bone. A micromanipulator was used to direct a glass pipette (Hirschmann microcapillary pipette, Z611239) of 20–30 μm internal tip diameter, based on coordinates established from the Paxinos mouse brain atlas^85^: 5.2 mm from Bregma, 39° angle from the vertical axis at the midline, 4.6 and 4.8 mm deep. One injection of 50–60 nl was performed at each depth with mechanical pressure (~10 nl/s). After each injection, the pipette was left in place for >10 min before being slowly withdrawn. Anterograde tracing was performed using AAV1-CAG-Flex-TdTomato (lot #AV5328A2) and rAAV5-CAGChR2- GFP (lot #AV4597D) at a 1:1 ratio, purchased from University of North Carolina (UNC) Vector Core. Retrograde tracing was performed with pAAVCAG- FLEX-rc [Jaws-KGC-GFP-ER2] (Addgene, Catalog No. 84445-AAVrg). Post-injection survival time for injected mice was 2–3 weeks.

### Genetically modified Rabies viral tracing

Stereotaxic surgery was performed as described above. On day one, *GAD67*^*Cre*^, *GlyT2*^*Cre*^, *VGluT2*^*Cre*^ and *SST*^*Cre*^ mice were injected with the helper AAV, either AAV1-EF1a-Flex-GTB (Salk Institute) or BA-96-AAV2/1-pAAV-Syn-Flex-nGToG-WPRE3 (Charité- Universitätsmedizin Berlin) in the vestibular structure or PrH. After 2–3 weeks of expression, either EnVA-G-deleted-Rabies-mCherry (Salk Institute) or BRABV-001-pSADB19dG-mCherry (Charité- Universitätsmedizin Berlin) were injected following the same coordinates. Post-injection survival time for rabies injected mice was 7 days for optimal trans-synaptic labelling with minimal toxicity.

### Ex vivo electrophysiology

For ex vivo recordings, 18 C57BL/6J mice, 3 *Kcng4*^Cre^;Ai27 mice and 1 *CaMKIIα*^*Cre/T29*^;Ai27 mouse from 30 to 60 days were deeply anesthetized with isoflurane and decapitated. As previously described^86^, the brain was quickly extracted and placed in ice-cold slice solution continuously carbogenated with 95% O2 and 5% CO2 containing the following (in mM): 240 Sucrose, 2.5 KCl, 1.25 NaH2PO4, 2 MgSO4, 1 CaCl2, 26 NaHCO3, 10 D-glucose. 250 μm thick coronal (for flocculus recordings) or sagittal (for vermis recordings) cerebellar slices were cut in ice-cold slice solution using a vibratome (VT1000S, Leica Biosystems, Wetzlar, Germany) with a ceramic blade (Campden Instruments Ltd, Manchester, United Kingdom). Slices were transferred to a recovery bath and incubated in continuously carbogenated artificial cerebrospinal fluid (ACSF) containing (in mM): 124 NaCl, 5 KCl, 1.25 Na2HPO4, 2 MgSO4, 2 CaCl2, 26 NaHCO3, 20 D-glucose and maintained at 34 °C for 1 h before being transferred to room temperature. For recording, individual slices were transferred to a recording chamber and maintained at 34 ± 1 °C with a feedback temperature controller (Scientifica, Uckfield, United Kingdom) under continuous perfusion with the carbogenated ACSF. For all recordings except *Kcng4*^Cre^; Ai27, slices were bathed with ACSF supplemented with synaptic receptor blockers, NMDA receptor antagonist D-AP5 (50 μM, Hellobio, Bristol, UK), selective and competitive AMPA receptor antagonist NBQX (10 μM, Hellobio, Bristol, UK), non-competitive GABAA receptor antagonist and glycine receptor inhibitor Picrotoxin (100 μM, Hellobio, Bristol, UK). PCs were visualized with SliceScope Pro 3000, a CCD camera, a trinocular eyepiece (Scientifica, Uckfield, UK) and Ocular (Teledyne Qimaging, Surrey, Canada). Recordings were obtained using borosilicate pipettes (Harvard apparatus, Holliston, MA, USA) with a resistance of 4–6 MΩ, filled with internal solution containing (in mM): 9 KCl, 3.48 MgCl2, 4 NaCl, 120 K + -Gluconate, 10 HEPES, 28.5 Sucrose, 4 Na2ATP, 0.4 Na3GTP in total pH 7.25–7.35, osmolarity 290–300 mOsmol/Kg (Sigma-Aldrich, Merck KGaA, Darmstadt, Germany) and 1 mg/ml biocytin. Recordings were performed using an ECP-10 amplifier (HEKA Electronics, Lambrecht, Germany) and digitized at 20 kHz. Optogenetic stimulation during *Kcng4*^Cre^; Ai27 recordings was induced using a pE-2 (CoolLED, Andover, UK) with LED wavelength at 470 nm and a 40× objective (Carl Zeiss). Recordings were excluded if the input resistance exceeded 25 MΩ. Acquisition was done in Patchmaster (HEKA Electronics, Lambrecht, Germany). Analysis was performed using a custom-build MATLAB code (Mathworks, Natick, MA, USA).

### In vivo electrophysiology

Mice underwent surgery to prepare for awake, head-fixed in vivo electrophysiological recordings of PCs. The same initial procedures were performed as in viral injections described above. After opening the scalp, the periosteum covering the skull and the muscle attachments over the interparietal bone were removed. Subsequently, a pedestal (a U-shaped holder for a small neodymium magnet (4 × 4 mm; MTG, Weilbach, Germany) was placed over the frontal and parietal bone for head-fixation by first applying a layer of Optibond (Kerr, Salerno, Italy) and then Charisma (Kulzer GmbH, Hanau, Germany). To target different areas in the cerebellum, a craniotomy (diameter ~3 mm) was made over the interparietal bone between the midline and lateral edge. A recording chamber was made around the craniotomy using Charisma, and closed with antibiotic ointment (Terra-Cortril+ polymyxine B; Eureco-Pharma B.V., Ridderkerk, The Netherlands) and silicone-glue (Picodent twinsil®; Dental-Produktions- und Vertriebs-GmbH, Wipperfürth, Germany) to protect the surface. Mice were given 2 days for recovery after surgery, followed by 2 days of acclimation to the recording setup. During recording, mice were head-fixed and walking on a wheel. Borosilicate glass pipettes capillaries (with filament, 1.5 OD, 0.86 ID; Sutter instrument, CA, USA) were pulled to obtain pipettes with a resistance of 5–10 MΩ at the tip and filled with a 2 M NaCl-solution. An electrode wire and optical fiber (200 µm × 50 mm, filter: 470 nm, driver: 20-1000 mA; LEDD1B; Thorlabs, Ely, UK) were placed inside the pipette solution, with the second to reach the start of the taper. Pipettes were controlled using a micromanipulator (MP-225; Sutter Instrument, CA, USA). PCs were opto-stimulated using a 50, 100 or 200 ms LED pulse with an intensity adjusted for an optimal response (constant amplitude during stimulation) to prevent depolarization block. Irradiance varied between 40 µW mm^−2^ and 300 µW mm^−2^, measured at the electrode tip (Optical Power Meter Model 1830-C; Newport Spectra-Physics BV, Utrecht). The signals were amplified (npi BA-03X; Science Products GmbH, Hofheim, Germany), filtered (CyberAmp 380, Axon; Molecular Devices, Sunnyvale, CA, USA), digitized (Micro3 1401; Cambridge Electronic Devices (CED), Cambridge, UK), and stored for offline analysis. To mark recording sites, iontophoretic injection of biotinylated dextran amine (BDA) (5%, Thermo-Fisher) was used with a constantly monitored anodal current of 1–8 µA, pulsed for 7 s during a period of 12–15 min (custom-made pulse generator).

Single-unit PCs were identified by the occurrence of simple spikes (SS) and complex spikes (CS) and a pause in SS after each CS (CF pause). Waveform clustering of Purkinje cell spiking activity was performed to identify SS and CS using Spike2 (CED, Cambridge, UK) and manually checked. Successful PC recordings included both the intrinsic firing of the PC and the LED triggered responses. Optogenetic stimulation epochs consisted of 0.5 s pre-stimulation time, a variable peri-stimulation time (50, 100 or 200 ms), and the remaining post-stimulation time to make up for a 2 s time window. The physiological properties were analyzed using a custom-written Python analysis script. For the baseline firing properties, the SS firing rate was determined. For the stimulation epochs, SS firing rate for the pre-stimulation, peri-stimulation and post-stimulation window were determined. The SS firing rate during peri-stimulation was then normalized to the SS firing rate during the pre-stimulation window. The average response of each cell was calculated by counting the mean number of SS events in a 5 ms bin over all single stimulations.

### Optogenetically-induced eye movement recordings and analysis

The surgical procedure was performed as described above with the addition of a cranial window for cannula placement. A flat-aligned skull was rotated 20° to provide access to the left periotic capsule. After the muscles over the periotic capsule were removed, a small craniotomy (diameter ~1 mm) was drilled 1 mm below the top of the capsule. A pipette tip (length 4 mm, tip diameter ~1 mm) was inserted with a 55° azimuth from the rostro-caudal midline and 13° elevation, fixed with dental cement (Simplex Rapid; Kemdent, Swindon, UK), and reinforced with Charisma.

During recording, mice (*Pcp2*^*Cre*^: *n* = 19; *Kcng4*^*Cre*^: *n* = 15; *CaMKIIα*^*Cre/T29*^: *n* = 8) were head-fixed and restrained in a custom-made plastic tube and placed on a turntable surrounded by a 360° drum with a random black-and-white squared pattern. Eye movements were recorded by video and calibrated (ETL-200, iScan; Illumina, San Diego, CA, USA). Three infrared emitters were used to illuminate the eye, so that the corneal reflection could be used as a reference point. An optical fiber (200 µm × 5 mm, filter: 470 nm, driver: 1000 µA, LEDD1B; Thorlabs, Ely, UK) was inserted into the cannula directed toward the flocculus. Irradiance was varied around 200 µW*mm^−2^ measured at the optic fiber tip. Signals were digitized and filtered (Power1401; CED, Cambridge, UK), and stored for offline analysis. For at rest data, the flocculus was opto-stimulated with the eye at rest (in absence of visual or vestibular input). The LED was turned on for 60 consecutive stimulations of 50, 100, 200, 500 and 1000 ms. For OKR, VOR, and VVOR co-stimulation data, optogenetic stimulation was either absent or provided during the naso-temporal portion or the temporo-nasal portion of the behavioral stimulation. Three additional *Kcng4*^*Cre*^;Ai27 mice were tested with decreasing light intensities with the intensity provided to the above mice set to 100%.

Eye movement position traces were analyzed with a custom MATLAB analysis script. The position of the eye is corrected and converted to angular degrees^87^. The eye position signal is differentiated and filtered to obtain a velocity signal, and this is repeated to obtain the corresponding acceleration signal. Fast eye movement components were automatically removed based on the calculated velocity signal (>40° s^−1^). Individual cycles were included if (1) the eye was at a steady position in the 20 ms prior to stimulation and (2) crossed a movement threshold (mean + 2 SD of the eye position 20 ms prior to stimulation). Included cycles were averaged to obtain the mean response per animal. Animal responses were averaged to obtain the average response per phenotype. The magnitude eye position was calculated using Pythagoras theorem (√(x^2^ + y^2^)). The normalized eye position was obtained by normalizing to the position of the eye 100 ms after LED onset. For visualization of the time course of statistical divergence between velocity profiles, the *t*-score was determined by conducting Unpaired two-tailed Student’s *t* test for each time point and the absolute value of the *t*-score is plotted as a color gradient. Based on the velocity profile during the maximum stimulus duration (1000 ms), the drive and release phase of the movement were set to 100 ms after onset or offset of the LED light, respectively. The hold phase is the time between the drive phase and LED offset, whereas the recovery phase is the first full second after the end of the release phase. A linear fit model was used to obtain the mean velocity and starting position of each movement phase. The net distance traveled was calculated with the area under the curve of the velocity profile of each segment.

### Statistics and reproducibility

Statistical analysis was performed with Prism GraphPad 9 (GraphPad Software Inc., San Diego CA, USA). All values are shown as mean ± SEM, unless otherwise stated. Statistical significance was calculated by one-way or two-way ANOVA (with repeated measures) with Holm-Šídák’s’s multiple comparisons test for normally distributed data, unless otherwise stated. In case of missing values, a mixed-effects analysis was performed. Non-normally distributed data was tested using Kruskal Wallis test with multiple comparisons, unless otherwise stated. A *p* value of <0.05 was considered significant. For immunohistological data, all experiments were replicated in at least three animals and included when results were consistent across replicates. Tests and exact *p* values are shown in Supplementary Table 1.

### Supplementary information

Supplementary Information

Supplementary movie 1

Supplementary movie 2

### Source data

Source data

## Data Availability

The data that support the findings of this study are available upon request from the corresponding author. Source data are provided with this paper.

## Code availability

The custom code that support the findings of this study are available upon request from the corresponding author.
