# Publisher Correction: Purkinje cell microzones mediate distinct kinematics of a single movement

**DOI:** 10.1038/s41467-023-40694-z

**Published:** 2023-09-04

**Authors:** François G. C. Blot, Joshua J. White, Amy van Hattem, Licia Scotti, Vaishnavi Balaji, Youri Adolfs, R. Jeroen Pasterkamp, Chris I. De Zeeuw, Martijn Schonewille

**Affiliations:** 1 Department of Neuroscience, Erasmus MC, Rotterdam, The Netherlands; 2 Department of Translational Neuroscience, University Medical Center Utrecht, Brain Center, Utrecht University, Utrecht, The Netherlands; 3 Netherlands Institute for Neuroscience, Royal Netherlands Academy of Arts and Sciences, Amsterdam, Netherlands

**Keywords:** Cerebellum, Reflexes, Neural circuits

Correction to: *Nature Communications* 10.1038/s41467-023-40111-5, published online 19 July 2023

The original version of the Article omitted Supplementary Movies 1 and 2. The HTML has been updated to include the Supplementary Movies.

