# Supplementary material for: Purkinje cell microzones mediate distinct kinematics of a single movement
Source: Nat Commun. 2023 Jul 19;14:4358. doi: 10.1038/s41467-023-40111-5 (PMC10356806; doi:10.1038/s41467-023-40111-5)
Supplement: Supplementary file 1 — Supplementary Information [file 41467_2023_40111_MOESM1_ESM.pdf]

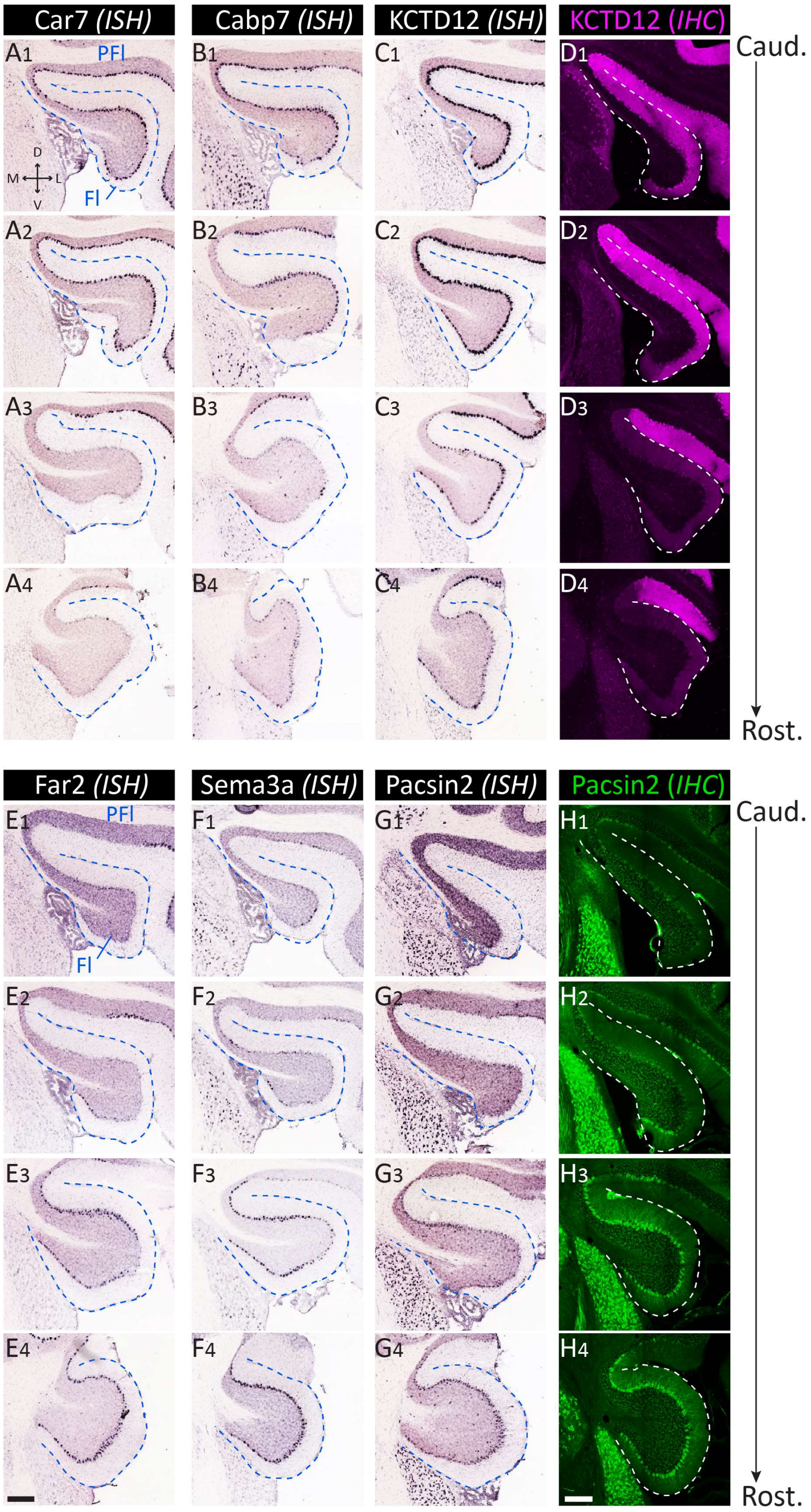

Fig. S1 In situ hybridization analysis demonstrates distinct regions of the mouse flocculus. (A-C & E-G) Serial images of in situ hybridization (ISH) on coronal sections of the flocculus (blue dashed line) from the Allen Brain Institute for Car7 (Exp. #74511736), Cabp7 (Exp. #73930835), KCTD12 (Exp. #73520993), Far2 (Exp. #71358652), Sema3a (Exp. #79591337), and Pacsin2 (Exp. #74047920). Section range from caudal (Caud.) to rostral (Rost.) (1→4). Scale Bar = 210 µm (applies to A-C and E-G) (D & H) Serial images of maximum intensity projections of confocal images of the flocculus (white dashed line) following immunohistochemistry (IHC) with anti-KCTD12 (D) or anti-Pacsin2 (H)

antibodies. Scale Bar = 200 μm (applies to D and H). Thickness = 40 μm; PFl=paraflocculus; Fl=flocculus; D=dorsal; V=ventral; M=medial; L=lateral.

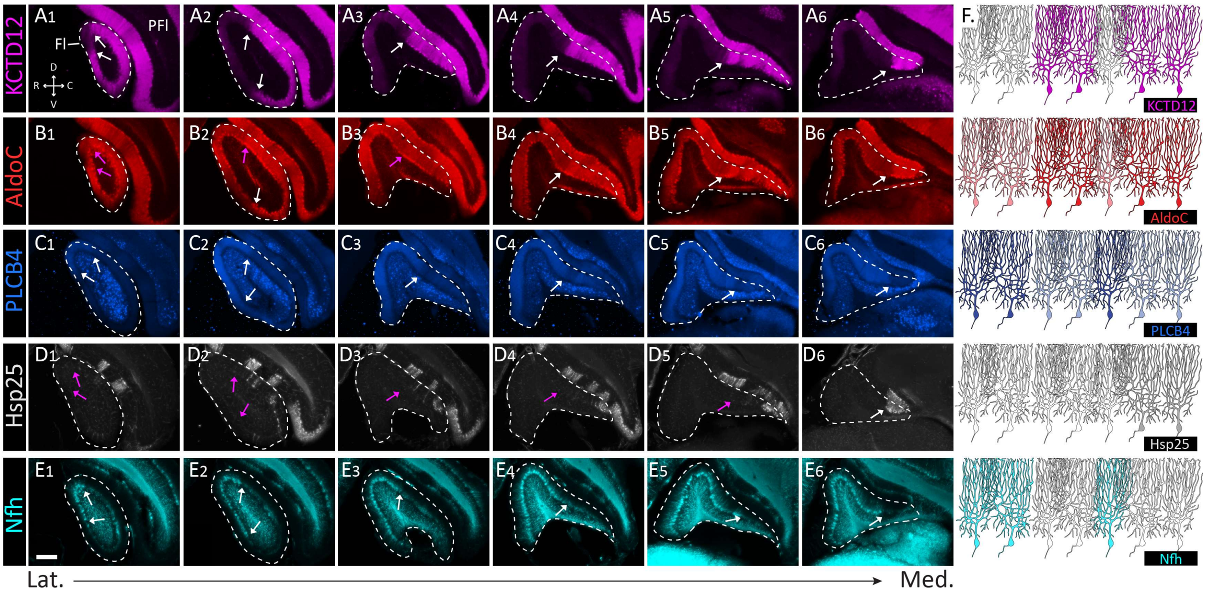

Fig. S2 Floccular compartmentations defined by KCTD12, AldoC, PLCB4, Hsp25 and Nfh. A-C, Serial sagittal sections of the flocculus (white dashed line) with KCTD12 (A), AldoC (B), and Hsp25 (C), immunolabeling. White arrows point at boundary identified in KCTD12 immunostaining and observed with other markers. Purple arrows point at boundaries identified in KCTD12 immunostaining but not clearly observable with other markers. Lateral (Lat.) to medial (Med.) (1→6). D, Schematic representation of differential protein expression among floccular PC subpopulations. PFl, paraflocculus; Fl, flocculus; C, caudal; R, rostral; D, dorsal; V, ventral. Scale bar = 200 μm (applies to all images).

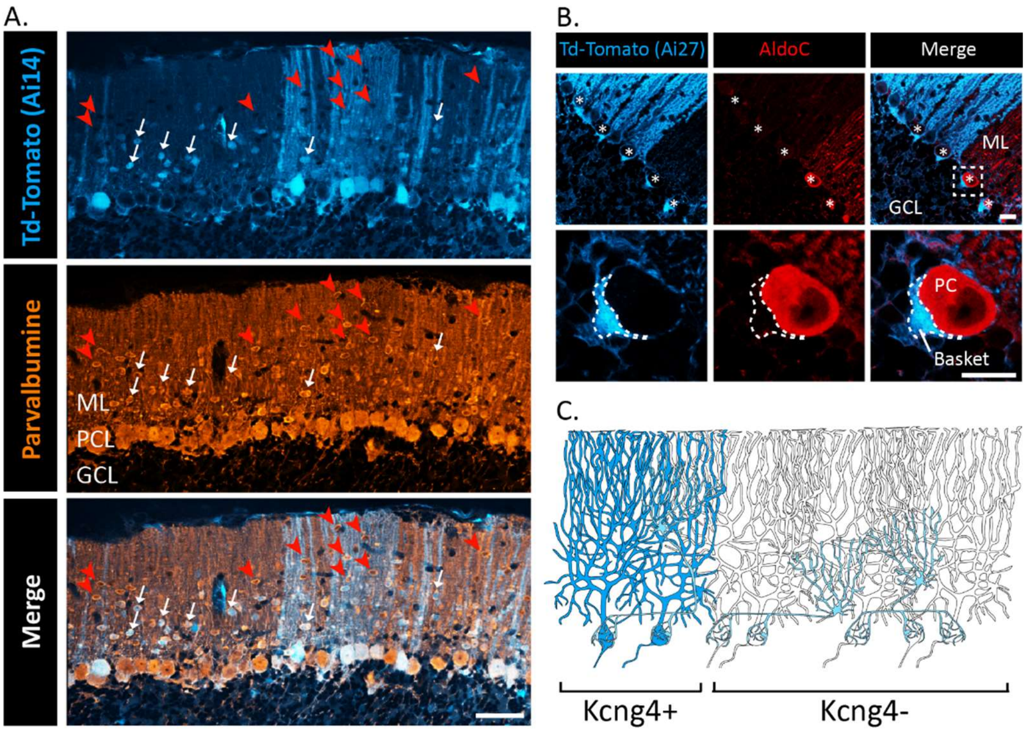

Fig. S3 *Kcng4*^{Cre} expression in AldoC- Purkinje cells and basket cells. A, Transverse section of the cerebellum of a *Kcng4*^{Cre}*;Ai14* mouse immunolabelled for parvalbumin. TdTomato expression under Cre recombinase activity labels PCs and parvalbumin+ interneurons, in the lower third of the molecular layer (white arrows). Parvalbumin+/TdTomato- interneurons localize to the upper part of the molecular layer (red arrow heads). Scale bar = 50 µm. B, Transverse section of the cerebellum of a *Kcng4*^{Cre}*;Ai27* mouse immunolabelled for AldoC. TdTomato expression reveals a basket-like structure around all PCs. Scale bars = 20 µm. C, Scheme of cell-type specific expression of Cre recombinase in the *Kcng4*^{Cre}. *Kcng4*- PCs are shown in white, *Kcng4*+ PCs are shown in blue and *Kcng4*+ baskets cells are shown in light blue.

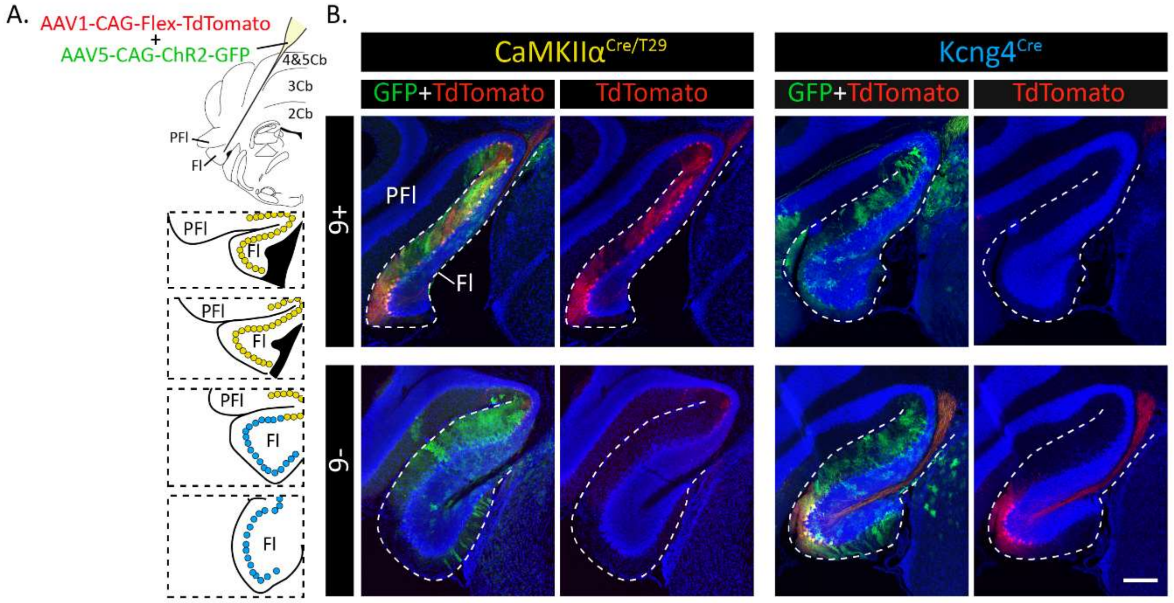

Fig. S4 Mouse lines allow for targeting of specific PC subpopulations in the flocculus. A, Scheme of the stereotaxic injection of AAV1-CAG-Flex-TdTomato/AAV5-CAG-ChR2-GFP in the flocculus with the expected location of Cre-expressing PCs in either $\textit{CaMKII}\alpha^{Cre/T29}$ (yellow dots) or $\textit{Kcng4}^{Cre}$ (blue dots) mice. B, Examples of injected flocculus in both models. GFP labeling (green) demarcates the injected region, TdTomato indicates the location of Cre-expressing Purkinje cells. Scale bar = 200 µm. Schematics adapted from Paxinos & Franklin, 2001[85].

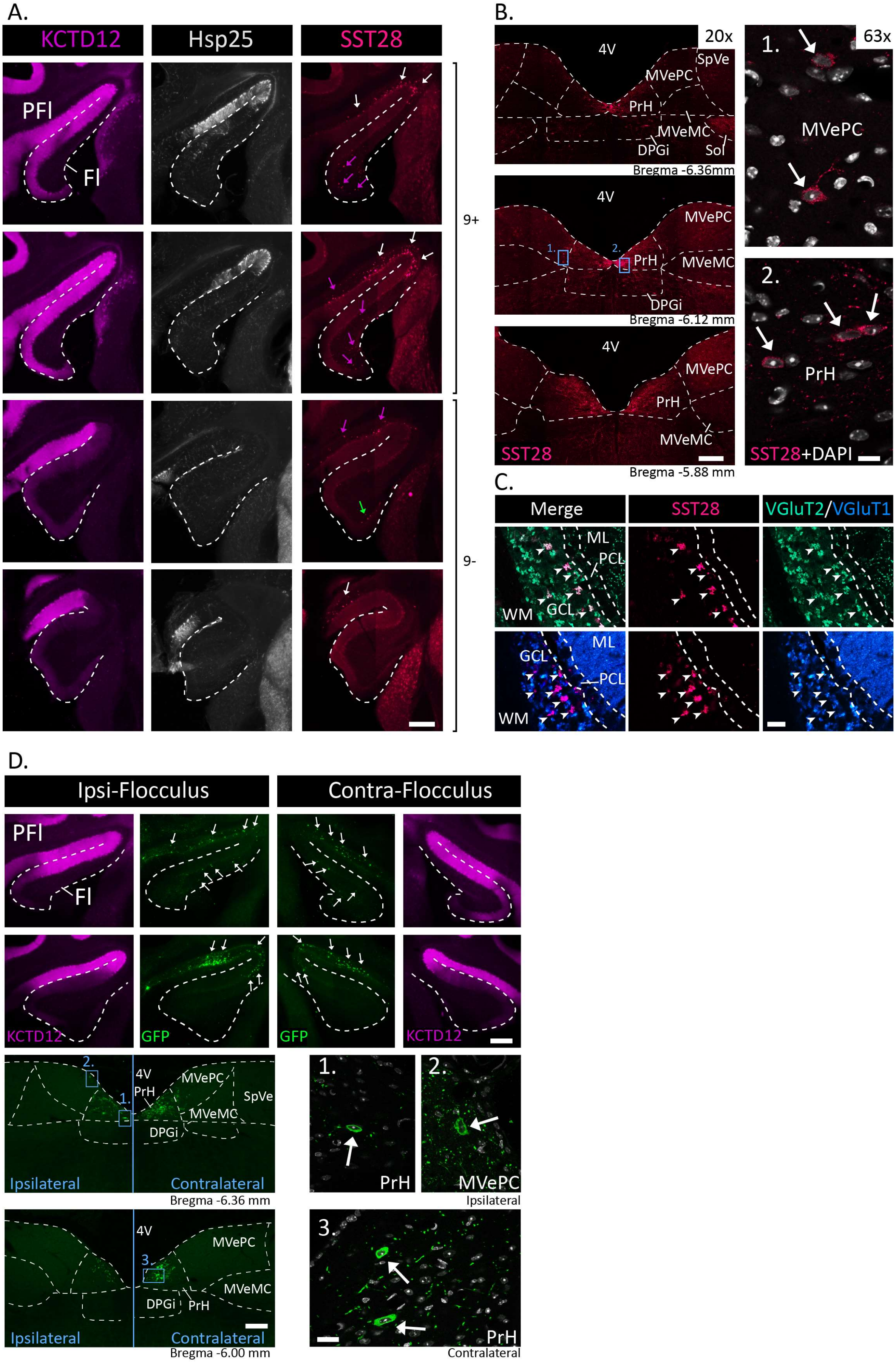
A.
KCTD12
Hsp25
SST28
PFl
Fl
9+
9-
B.
20x
63x
4V
SpVe
MVePC
PrH
MVeMC
DPGi
Sol
Bregma -6.36mm
Bregma -6.12 mm
Bregma -5.88 mm
SST28
SST28+DAPI
C.
Merge
SST28
VGluT2/VGluT1
ML
PCL
GCL
WM
D.
Ipsi-Flocculus
Contra-Flocculus
GFP
KCTD12
Ipsilateral
Contralateral
Bregma -6.00 mm

Fig. S5 SST expression delineates mossy fibers that target the 9+ (KCTD12+) region of the flocculus. A) Serial transverse sections of the flocculus (white dashed line) with KCTD12, Hsp25, and SST28 immunolabeling. SST28+ MFs are present in Hsp25+ domain (white arrows), KCTD12+ domain (white + purple arrows) and KCTD12- domain (green arrow). Scale Bar = 200μm. (B) SST28 immunoreactivity in the dorsal brainstem, with SST28+ cells in the MVePC (1.) and the Pr (2.). Scale Bar 20x = 200μm. Scale Bar 63x = 10μm. (C) Characterization of SST28+ MFs molecular identity with co-labeling with VGluT1 or VGluT2 vesicular transporters. Scale Bar = 20μm. (D) Unilateral injection of retrograde AAV (pAAV-CAG-FLEX-rc [Jaws-KGC-GFP-ER2]) in the flocculus of SST^{Cre} mice showed labelled MFs restricted to the KCTD12+ domain in both ipsi- and contralateral flocculus, and retrogradelly labeled neurons in ipsi- and contralateral Pr (1. And 3.) and MVePC (2.). Scale Bar for flocculus = 200μm; Scale Bar for brainstem(20x) = 200μm; Scale Bar for brainstem(63x) = 20μm. PFl=paraflocculus; Fl=flocculus; PrH=nucleus prepositus hypoglossi; MVePC=medial vestibular parvicellular; MVeMC=medial vestibular magnocellular; SpVe=spinal vestibular; DPGi=dorsal paragigantocellular nucleus of the reticular formation; Sol=solitary nucleus; 4V=fourth ventricle; ML=molecular layer; PCL=Purkinje cell layer; GCL= granular cell layer; C=caudal; R=rostral; D=dorsal; V=ventral.

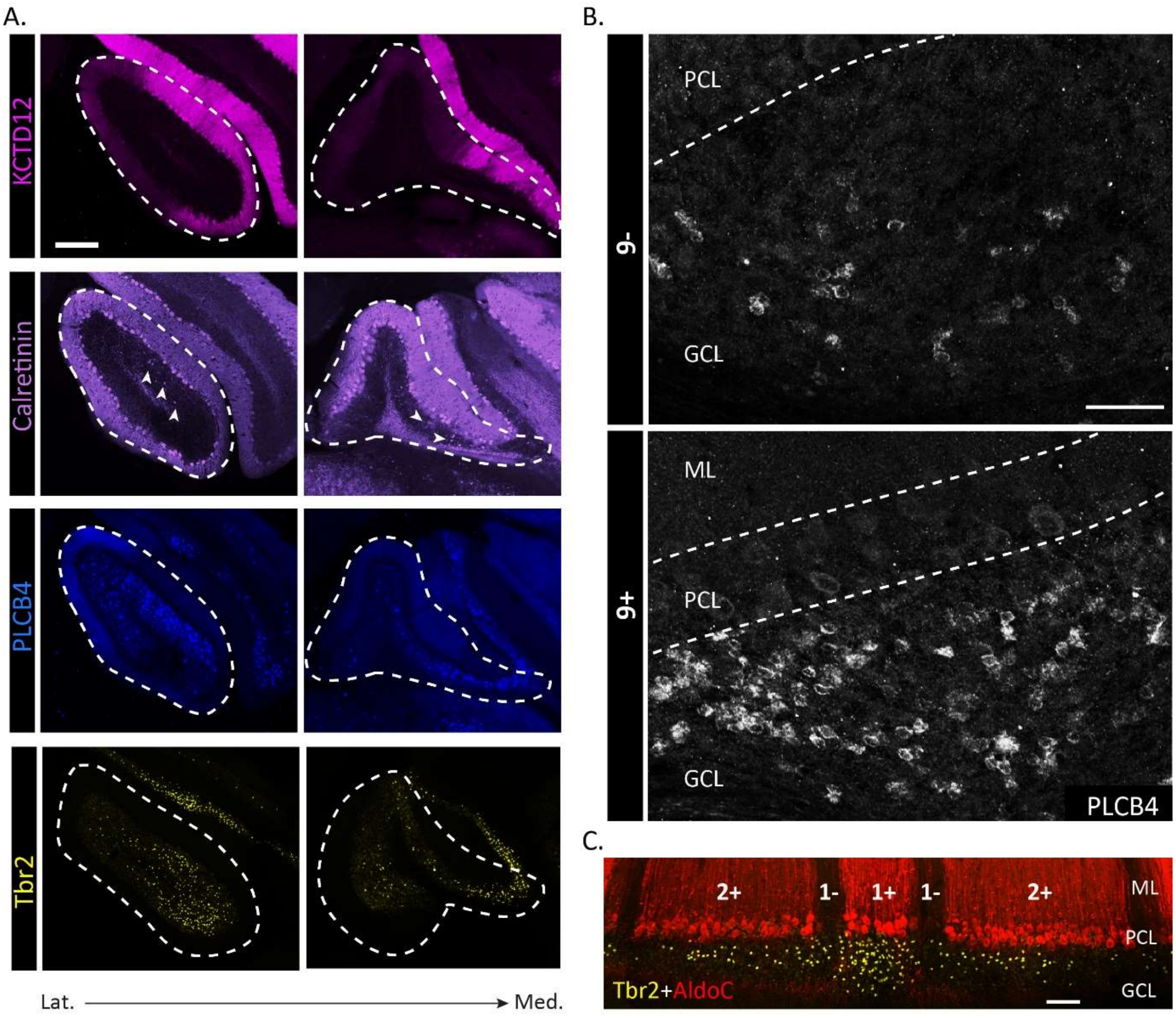

Fig. S6 UBC distribution in the flocculus. A, Sagittal sections of the lateral (Lat.) and medial (Med.) flocculus (outlined with a white dashed line) with KCTD12, Calretinin, PLCB4 and Tbr2 immunolabeling. White arrow heads point to sparse Calrt+ UBCs. Scale bar = 200 µm. B, High magnification images of the PLCB4+ UBC population below the 9- and 9+ PC subpopulations. Scale bar = 50 µm. C, Overview of UBC (Tbr2+) distribution in lobule IX relative to the AldoC compartments. Scale bar = 100 µm.

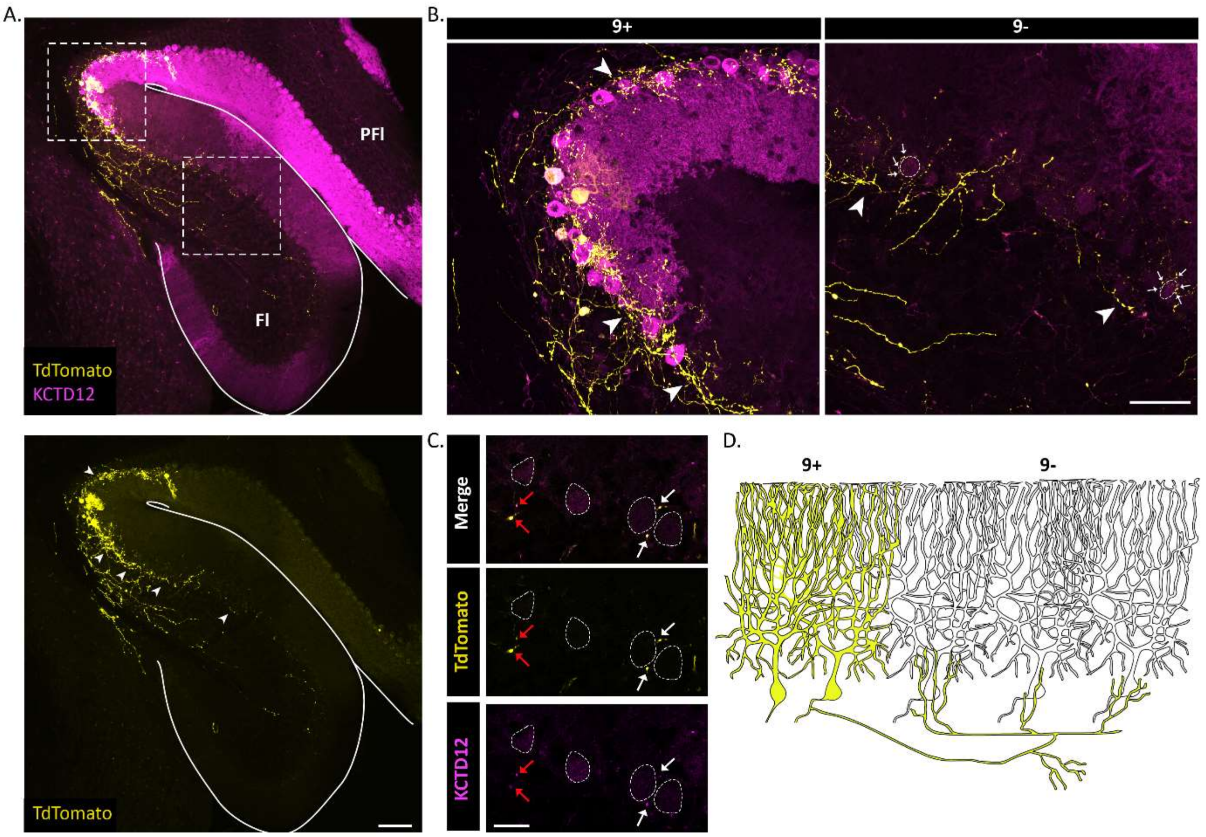

Fig. S7 PC collaterals interconnect subpopulations. A, Injection of AAV1-CAG-Flex-TdTomato (yellow) in the flocculus of *CaMKIIα*$^{Cre/T29}$ mice, with KCTD12 (purple) immunolabeling. White arrow heads point to PC collaterals spreading in the PC layer, into an area also known as the infraganglionic plexus. Scale bar = 100 µm. B, High magnification images of labelled 9+ PC collaterals below 9+ (left) and 9- (right) PC subpopulations. White arrows point to bouton-like structures onto 9- PCs (dashed circles). Scale bar = 50 µm. C, Identification of TdTomato+/KCTD12+ boutons onto 9- PCs (white arrows) or below (red arrows), in the granular cell layer. Scale bar = 20 µm. D, Schematic representation of PC collaterals from one subpopulation connecting another.

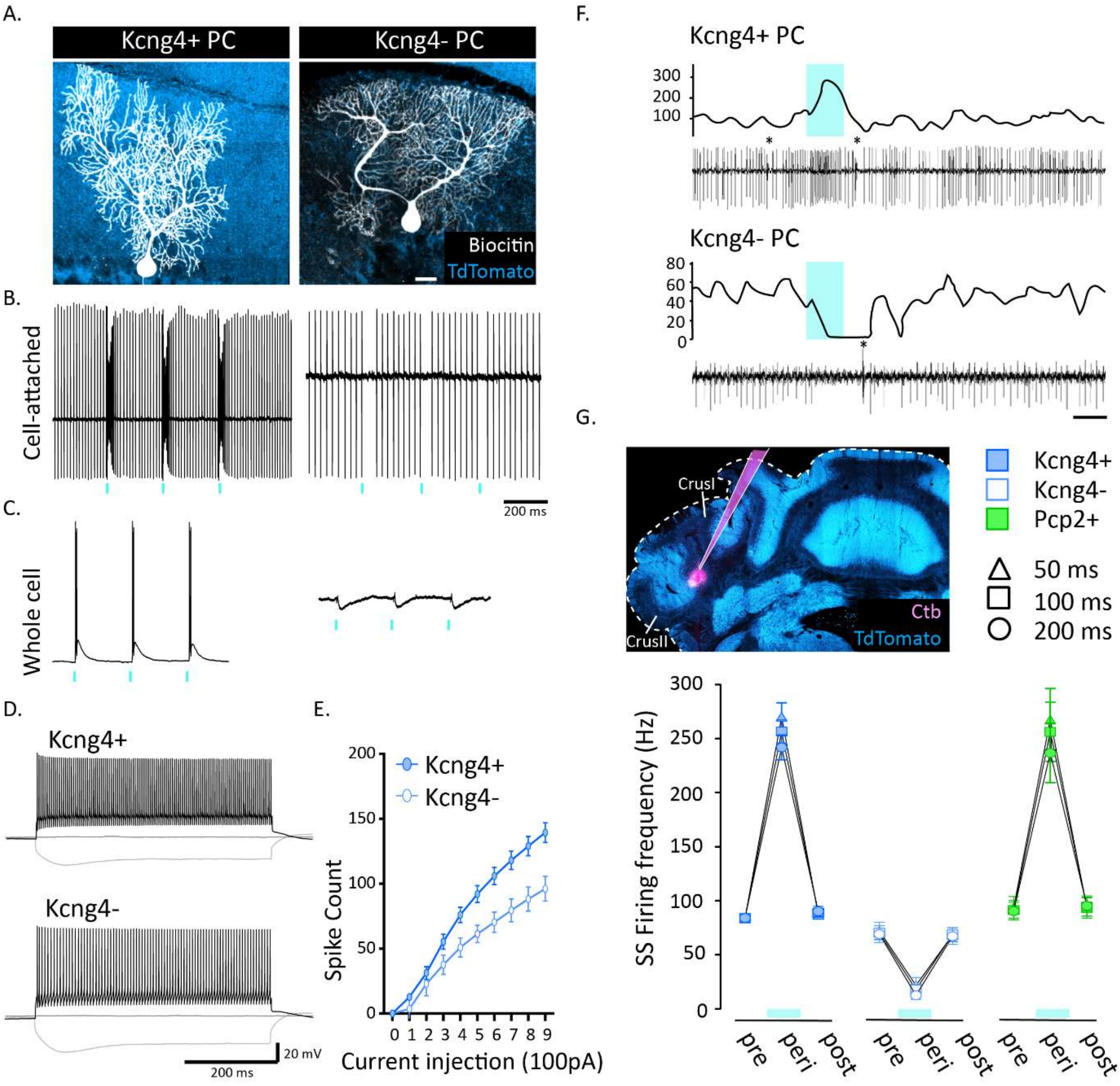

Fig. S8 Effects of optogenetic stimulation in *Kcng4*^{Cre};Ai14 Purkinje cells and basket cells. A, Example biotin-filled *Kcng4*+ (left) and *Kcng4*- (right) Purkinje cells. Scale bar = 20 μm. B, Example cell-attached recordings with 5-ms optogenetic stimulations (blue bars) from *Kcng4*+ (left) and *Kcng4*- (right). C, Example whole cell recordings with 5-ms optogenetic stimulations (blue bars) from *Kcng4*+ (left) and *Kcng4*- (right). D, Example current injection step responses during whole cell recordings from *Kcng4*+ (top) and *Kcng4*- (bottom). Responses to -600, 0, and 900 pA current injections for 500 ms from holding current are shown. E, Quantification of the number of action potentials induced at different 500-ms positive current injection steps in *Kcng4*+ and *Kcng4*- PCs. F, Example in vivo recordings with optogenetic stimulation in *Kcng4*+ and *Kcng4*- PCs. Scale bar = 100 ms. G, Example labeled recording site with biotin examined with immunohistochemistry. K, quantification of pre, peri, and post-stimulus simple spike (SS) firing frequency with optogenetic stimulation. ML = molecular layer; PCL = Purkinje cell layer; GCL = granular cell layer. All data are presented as mean values +/- SEM.

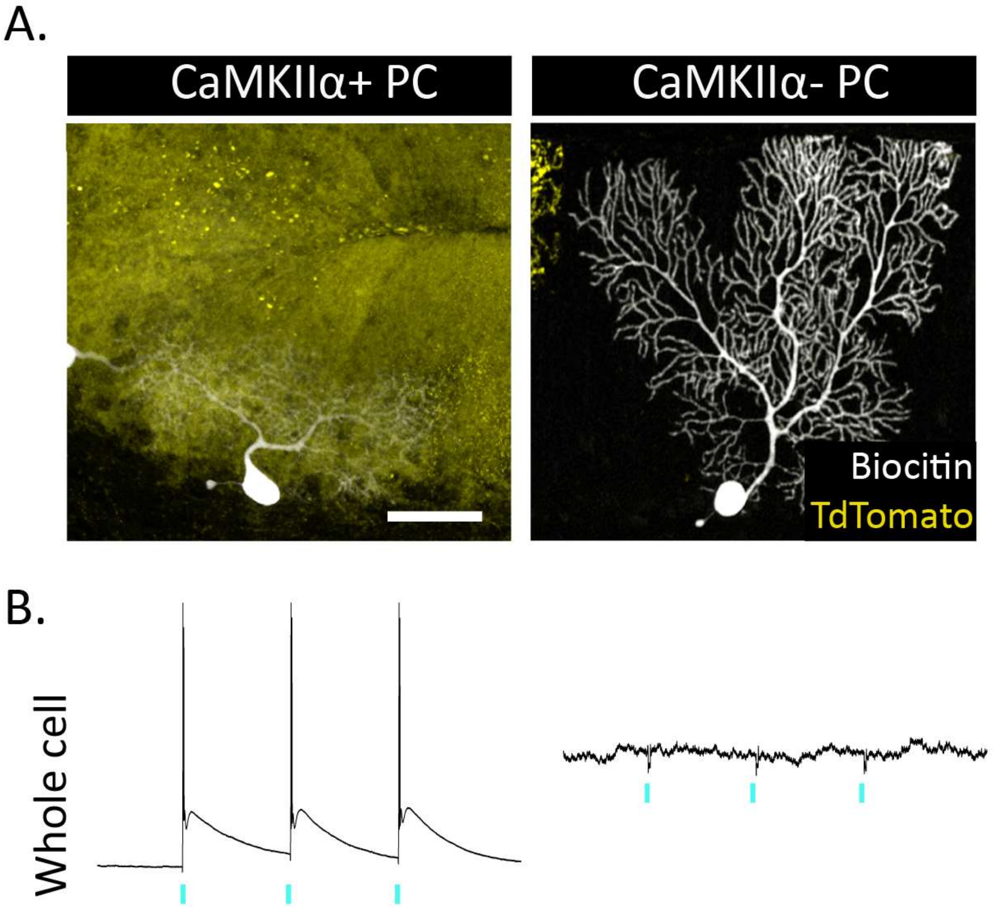

Fig. S9 Effects of optogenetic stimulation in *CaMKIIα*$^{Cre/T29}$ x Ai14 Purkinje cells. A, Example biotin-filled *CaMKIIα*$^{Cre/T29}$+ (left) and *CaMKIIα*$^{Cre/T29}$- (right) Purkinje cells. Scale bar = 20 μm. B, Example whole cell recordings with 5-ms optogenetic stimulations (blue bars) from *CaMKIIα*$^{Cre/T29}$+ (left) and *CaMKIIα*$^{Cre/T29}$- (right).

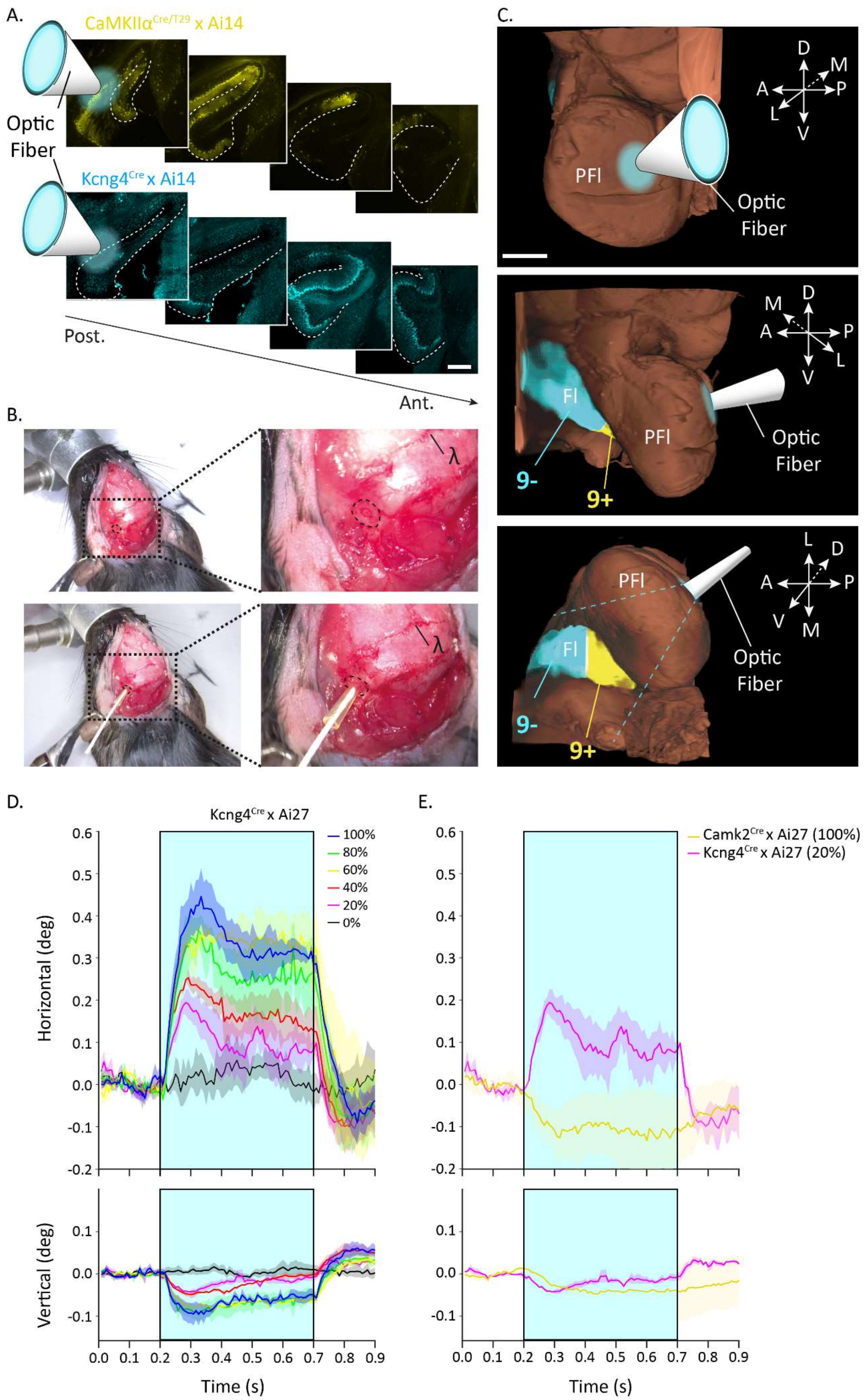

A.
CaMKIIα$^{Cre/T29}$ x Ai14
Optic Fiber
Kcng4^{Cre} x Ai14
Post.
Ant.
B.
λ
C.
D
M
A
P
L
V
PFl
Optic Fiber
Fl
9-
9+
D.
Kcng4^{Cre} x Ai27
100%
80%
60%
40%
20%
0%
Horizontal (deg)
Vertical (deg)
Time (s)
E.
Camk2^{Cre} x Ai27 (100%)
Kcng4^{Cre} x Ai27 (20%)
Time (s)

Fig. S10 Position of the optic fiber for optogenetic stimulation of the flocculus. A, Position of the source of optogenetic light (optic fiber) relative to PC subpopulations observed in *CaMKIIα*$^{Cre/T29}$;Ai14 (yellow) and *Kcng4*^{Cre};Ai14 (blue) mice. Scale bar = 200 µm. B, Surgical implantation of the canula over the paraflocculus (dashed circle). A paper tip illustrates the position of the optic fiber. C, 3D reconstruction based on the analysis of an iDISCO-cleared and lightsheet-imaged brain (Suppl. Video 1) of the flocculus and paraflocculus to illustrate the relative distance between the optic fibers and the PC subpopulations. Scale bar = 500 µm. PFl, paraflocculus; Fl, flocculus; A, anterior; P, posterior; D, dorsal; V, ventral; L, lateral; M, medial. D, Means of horizontal (top) and vertical (bottom) eye movement during stepwise optogenetic stimulation (500ms, blue rectangles) on *Kcng4*^{Cre}*;Ai27*, lowering intensity of illumination. E, Means of horizontal (top) and vertical (bottom) eye movement during optogenetic stimulation of *CaMKIIα*$^{Cre/T29}$*;Ai27* with full power of illumination (see Fig. 5) compared to *Kcng4*^{Cre}*;Ai27* mice illuminated with 20% of the full power of illumination. All data are presented as mean values +/- SEM.

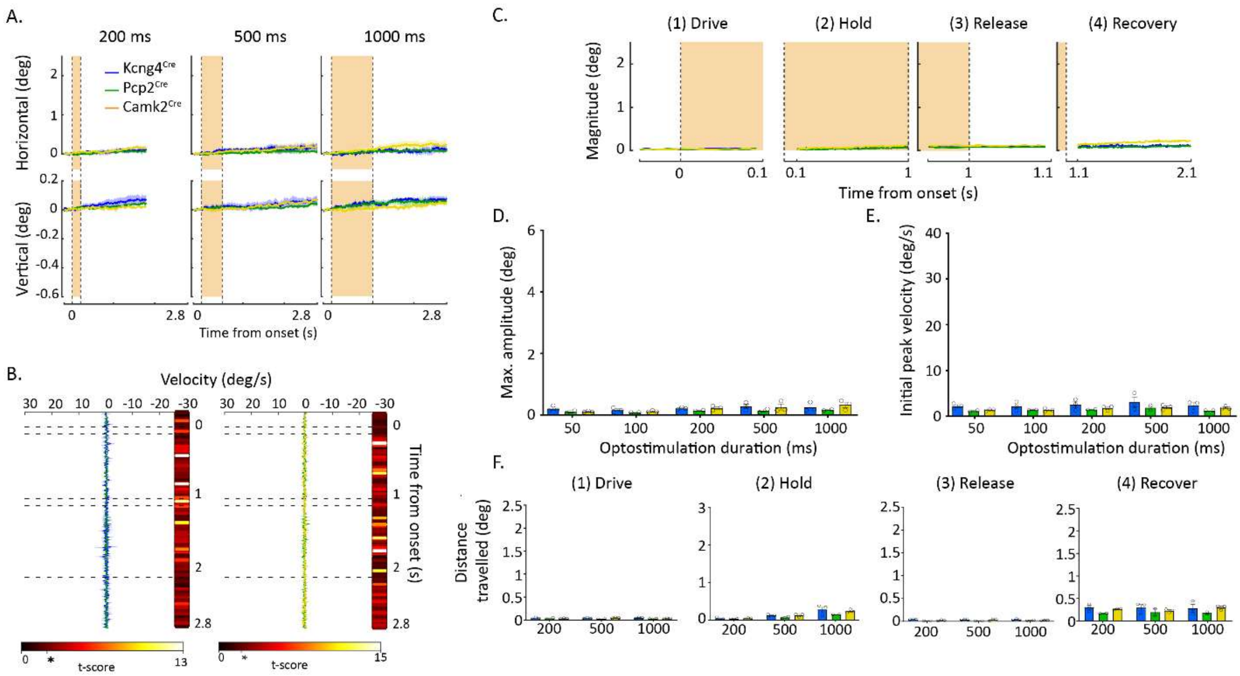

Fig. S11 Optogenetic inhibition of Purkinje cells in the flocculus has little to no ability to drive eye movement regardless of genotype. A, Means of horizontal (top) and vertical (bottom) eye movement traces for *Pcp2*^{Cre}*;Ai39* (green), *Kcng4*^{Cre}*;Ai39* (blue), and *CaMKIIα*$^{Cre/T29}$*;Ai39* (yellow) mice. Optogenetic stimulation durations are 200, 500 or 1000 ms. B, Velocity profiles of mean magnitude eye movement response during 1000 ms optogenetic stimulation. The velocity profile shows a peak after LED onset and LED offset. The heatmap shows the statistical divergence in velocity at each time point using the t-score from the unpaired two-tailed Student's t test. Dotted lines indicate movement phases based on peak velocity. C, Representation of the four phases (1) drive, (2) hold, (3) release, and (4) recovery obtained from the velocity traces, displaying the absolute magnitude of the movement for optogenetic stimulation of 1000 ms. D, Maximum amplitude of the eye movement response reached at the end of optogenetic stimulation for all optogenetic stimulation durations. E, Initial peak velocity of the magnitude eye movement response occurring shortly after LED onset. F, Distance traveled by the eye during the four phases of the induced eye movement. All data are presented as mean values +/- SEM. n = 3 *Kcng4*^{Cre}*;Ai39* mice, 2 *Pcp2*^{Cre}*;Ai39* mice and 3 *CaMKIIα*$^{Cre/T29}$*;Ai39* mice.

Supplementary Video1. Cleared *Kcng4*^{Cre} x Ai14 brain with iDISCO protocol. Light sheet imaging in the coronal orientation shows the distribution of 9- PCs (0:04 to 0:11) and 9+ PCs (0:12 to 0:18). The 3D reconstruction illustrates the distribution of both sub-populations in the structure of the flocculus (red). The model was used to locate 9+ (yellow) and 9- (blue) sub-populations relative to the optic fiber used for optogenetic placed over the posterior segment of the paraflocculus. Related to **Fig. 2** and **Fig. S8**

Supplementary Video2. Cleared *Kcng4*^{Cre} x Ai14 brain (left) and wild type brain (right) with iDISCO protocol. The wild type brain was labelled for Tbr2 (yellow), SST (pink), and NECAB1 (white) to mark respectively UBCs, mossy fibers and BIN cells. Light sheet imaging in the coronal orientation shows the distribution of UBCs, SST+ mossy fibers and BIN cells from posterior to anterior relative to 9+ PCs (0:04 to 1:33) and 9+ PCs (1:34 to 2:00) observed in the *Kcng4*^{Cre} x Ai14 brain. 3D reconstruction of confocal images from standard immunostaining protocol shows the density of UBCs and SST+ mossy fibers in the flocculus. Calretinin immunolabelling (mauve) illustrates the characteristic shape of UBCs, while they remain sparse in the flocculus. SST+ mossy fibers are all VGluT2+ (green). Related to **Fig. S5** and **S6**

Supplementary table 1

Figure	Groups compared	N	Significance
The following data were tested with a one-way ANOVA with multiple comparisons.			
Fig 1F Firing Rate	III vs X	14 vs 35	<0.0001
Fig 1F Firing Rate	III vs 9-	14 vs 20	0.0124
Fig 1F Firing Rate	III vs 9+	14 vs 14	<0.0001
Fig 1F Firing Rate	X vs 9-	35 vs 20	0.0944
Fig 1F Firing Rate	X vs 9+	35 vs 14	0.8093
Fig 1F Firing Rate	9- vs 9+	20 vs 14	0.0294
Fig 1F CV2	III vs X	14 vs 35	0.0022
Fig 1F CV2	III vs 9-	14 vs 20	0.0301
Fig 1F CV2	III vs 9+	14 vs 14	0.0094
Fig 1F CV2	X vs 9-	35 vs 20	0.8338
Fig 1F CV2	X vs 9+	35 vs 14	>0.9999
Fig 1F CV2	9- vs 9+	20 vs 14	0.8771
Fig 1H Spike Count	III vs X	20 vs 51	0.0064
Fig 1H Spike Count	III vs 9-	20 vs 32	0.0079
Fig 1H Spike Count	III vs 9+	20 vs 36	0.0025
Fig 1H Spike Count	X vs 9-	51 vs 32	0.0040
Fig 1H Spike Count	X vs 9+	51 vs 36	0.0003
Fig 1H Spike Count	9- vs 9+	32 vs 36	0.0006
The following data were tested with a two-way ANOVA with multiple comparisons.			
Fig 1H Time to first (step one)	III vs X	20 vs 51	<0.0001
Fig 1H Time to first (step one)	III vs 9-	20 vs 32	<0.0001
Fig 1H Time to first (step one)	III vs 9+	20 vs 36	0.0045
Fig 1H Time to first (step one)	X vs 9-	51 vs 32	0.4192
Fig 1H Time to first (step one)	X vs 9+	51 vs 36	0.0040
Fig 1H Time to first (step one)	9- vs 9+	32 vs 36	0.3676
Fig 1H Time to first (step two)	III vs X	20 vs 51	0.1410
Fig 1H Time to first (step two)	III vs 9-	20 vs 32	0.9961
Fig 1H Time to first (step two)	III vs 9+	20 vs 36	0.0001
Fig 1H Time to first (step two)	X vs 9-	51 vs 32	0.1175
Fig 1H Time to first (step two)	X vs 9+	51 vs 36	0.0179
Fig 1H Time to first (step two)	9- vs 9+	32 vs 36	<0.0001
Fig 5E Max. amp. (50)	Kcng4 vs Pcp2	15 vs 19	>0.99
Fig 5E Max. amp. (50)	Kcng4 vs Camk2	15 vs 8	<0.001
Fig 5E Max. amp. (50)	Pcp2 vs Camk2	19 vs 8	<0.001
Fig 5E Max. amp. (100)	Kcng4 vs Pcp2	15 vs 19	0.87
Fig 5E Max. amp. (100)	Kcng4 vs Camk2	15 vs 8	0.002
Fig 5E Max. amp. (100)	Pcp2 vs Camk2	19 vs 8	<0.001
Fig 5E Max. amp. (200)	Kcng4 vs Pcp2	15 vs 19	0.68
Fig 5E Max. amp. (200)	Kcng4 vs Camk2	15 vs 8	0.09
Fig 5E Max. amp. (200)	Pcp2 vs Camk2	19 vs 8	0.009
Fig 5E Max. amp. (500)	Kcng4 vs Pcp2	15 vs 19	0.33
Fig 5E Max. amp. (500)	Kcng4 vs Camk2	15 vs 8	0.36
Fig 5E Max. amp. (500)	Pcp2 vs Camk2	19 vs 8	0.02

Fig 5E Max. amp. (1000)	Kcng4 vs Pcp2	15 vs 19	0.15
Fig 5E Max. amp. (1000)	Kcng4 vs Camk2	15 vs 8	0.01
Fig 5E Max. amp. (1000)	Pcp2 vs Camk2	19 vs 8	0.001
Fig 5F Init. Peak Vel. (50)	Kcng4 vs Pcp2	15 vs 19	>0.99
Fig 5F Init. Peak Vel. (50)	Kcng4 vs Camk2	15 vs 8	<0.001
Fig 5F Init. Peak Vel. (50)	Pcp2 vs Camk2	19 vs 8	<0.001
Fig 5F Init. Peak Vel. (100)	Kcng4 vs Pcp2	15 vs 19	>0.99
Fig 5F Init. Peak Vel. (100)	Kcng4 vs Camk2	15 vs 8	<0.001
Fig 5F Init. Peak Vel. (100)	Pcp2 vs Camk2	19 vs 8	<0.001
Fig 5F Init. Peak Vel. (200)	Kcng4 vs Pcp2	15 vs 19	>0.99
Fig 5F Init. Peak Vel. (200)	Kcng4 vs Camk2	15 vs 8	<0.001
Fig 5F Init. Peak Vel. (200)	Pcp2 vs Camk2	19 vs 8	<0.001
Fig 5F Init. Peak Vel. (500)	Kcng4 vs Pcp2	15 vs 19	>0.99
Fig 5F Init. Peak Vel. (500)	Kcng4 vs Camk2	15 vs 8	<0.001
Fig 5F Init. Peak Vel. (500)	Pcp2 vs Camk2	19 vs 8	<0.001
Fig 5F Init. Peak Vel. (1000)	Kcng4 vs Pcp2	15 vs 19	>0.99
Fig 5F Init. Peak Vel. (1000)	Kcng4 vs Camk2	15 vs 8	<0.001
Fig 5F Init. Peak Vel. (1000)	Pcp2 vs Camk2	19 vs 8	<0.001
Fig 5G Abs. Mean Vel. (Drive, 200)	Kcng4 vs Pcp2	15 vs 19	0.83
Fig 5G Abs. Mean Vel. (Drive, 200)	Kcng4 vs Camk2	15 vs 8	<0.001
Fig 5G Abs. Mean Vel. (Drive, 200)	Pcp2 vs Camk2	19 vs 8	<0.001
Fig 5G Abs. Mean Vel. (Drive, 500)	Kcng4 vs Pcp2	15 vs 19	0.78
Fig 5G Abs. Mean Vel. (Drive, 500)	Kcng4 vs Camk2	15 vs 8	<0.001
Fig 5G Abs. Mean Vel. (Drive, 500)	Pcp2 vs Camk2	19 vs 8	<0.001
Fig 5G Abs. Mean Vel. (Drive, 1000)	Kcng4 vs Pcp2	15 vs 19	0.74
Fig 5G Abs. Mean Vel. (Drive, 1000)	Kcng4 vs Camk2	15 vs 8	<0.001
Fig 5G Abs. Mean Vel. (Drive, 1000)	Pcp2 vs Camk2	19 vs 8	<0.001
Fig 5G Abs. Mean Vel. (Hold, 200)	Kcng4 vs Pcp2	15 vs 19	0.29
Fig 5G Abs. Mean Vel. (Hold, 200)	Kcng4 vs Camk2	15 vs 8	0.73
Fig 5G Abs. Mean Vel. (Hold, 200)	Pcp2 vs Camk2	19 vs 8	0.08
Fig 5G Abs. Mean Vel. (Hold, 500)	Kcng4 vs Pcp2	15 vs 19	0.02
Fig 5G Abs. Mean Vel. (Hold, 500)	Kcng4 vs Camk2	15 vs 8	0.11
Fig 5G Abs. Mean Vel. (Hold, 500)	Pcp2 vs Camk2	19 vs 8	0.003
Fig 5G Abs. Mean Vel. (Hold, 1000)	Kcng4 vs Pcp2	15 vs 19	0.007
Fig 5G Abs. Mean Vel. (Hold, 1000)	Kcng4 vs Camk2	15 vs 8	0.04
Fig 5G Abs. Mean Vel. (Hold, 1000)	Pcp2 vs Camk2	19 vs 8	<0.001
Fig 5G Abs. Mean Vel. (Release, 200)	Kcng4 vs Pcp2	15 vs 19	0.85
Fig 5G Abs. Mean Vel. (Release, 200)	Kcng4 vs Camk2	15 vs 8	<0.001
Fig 5G Abs. Mean Vel. (Release, 200)	Pcp2 vs Camk2	19 vs 8	<0.001
Fig 5G Abs. Mean Vel. (Release, 500)	Kcng4 vs Pcp2	15 vs 19	0.90
Fig 5G Abs. Mean Vel. (Release, 500)	Kcng4 vs Camk2	15 vs 8	<0.001
Fig 5G Abs. Mean Vel. (Release, 500)	Pcp2 vs Camk2	19 vs 8	<0.001
Fig 5G Abs. Mean Vel. (Release, 1000)	Kcng4 vs Pcp2	15 vs 19	0.85
Fig 5G Abs. Mean Vel. (Release, 1000)	Kcng4 vs Camk2	15 vs 8	<0.001
Fig 5G Abs. Mean Vel. (Release, 1000)	Pcp2 vs Camk2	19 vs 8	<0.001
Fig 5G Abs. Mean Vel. (Recover, 200)	Kcng4 vs Pcp2	15 vs 19	0.03
Fig 5G Abs. Mean Vel. (Recover, 200)	Kcng4 vs Camk2	15 vs 8	0.96
Fig 5G Abs. Mean Vel. (Recover, 200)	Pcp2 vs Camk2	19 vs 8	0.91
Fig 5G Abs. Mean Vel. (Recover, 500)	Kcng4 vs Pcp2	15 vs 19	0.01
Fig 5G Abs. Mean Vel. (Recover, 500)	Kcng4 vs Camk2	15 vs 8	>0.99

Fig 5G Abs. Mean Vel. (Recover, 500)	Pcp2 vs Camk2	19 vs 8	0.63
Fig 5G Abs. Mean Vel. (Recover, 1000)	Kcng4 vs Pcp2	15 vs 19	0.02
Fig 5G Abs. Mean Vel. (Recover, 1000)	Kcng4 vs Camk2	15 vs 8	>0.99
Fig 5G Abs. Mean Vel. (Recover, 1000)	Pcp2 vs Camk2	19 vs 8	0.23
Fig 5H Dist. Travelled (Drive, 200)	Kcng4 vs Pcp2	15 vs 19	0.80
Fig 5H Dist. Travelled (Drive, 200)	Kcng4 vs Camk2	15 vs 8	<0.001
Fig 5H Dist. Travelled (Drive, 200)	Pcp2 vs Camk2	19 vs 8	<0.001
Fig 5H Dist. Travelled (Drive, 500)	Kcng4 vs Pcp2	15 vs 19	0.75
Fig 5H Dist. Travelled (Drive, 500)	Kcng4 vs Camk2	15 vs 8	<0.001
Fig 5H Dist. Travelled (Drive, 500)	Pcp2 vs Camk2	19 vs 8	<0.001
Fig 5H Dist. Travelled (Drive, 1000)	Kcng4 vs Pcp2	15 vs 19	0.70
Fig 5H Dist. Travelled (Drive, 1000)	Kcng4 vs Camk2	15 vs 8	<0.001
Fig 5H Dist. Travelled (Drive, 1000)	Pcp2 vs Camk2	19 vs 8	<0.001
Fig 5H Dist. Travelled (Hold, 200)	Kcng4 vs Pcp2	15 vs 19	0.22
Fig 5H Dist. Travelled (Hold, 200)	Kcng4 vs Camk2	15 vs 8	0.08
Fig 5H Dist. Travelled (Hold, 200)	Pcp2 vs Camk2	19 vs 8	0.02
Fig 5H Dist. Travelled (Hold, 500)	Kcng4 vs Pcp2	15 vs 19	0.07
Fig 5H Dist. Travelled (Hold, 500)	Kcng4 vs Camk2	15 vs 8	>0.99
Fig 5H Dist. Travelled (Hold, 500)	Pcp2 vs Camk2	19 vs 8	0.04
Fig 5H Dist. Travelled (Hold, 1000)	Kcng4 vs Pcp2	15 vs 19	0.04
Fig 5H Dist. Travelled (Hold, 1000)	Kcng4 vs Camk2	15 vs 8	0.97
Fig 5H Dist. Travelled (Hold, 1000)	Pcp2 vs Camk2	19 vs 8	0.02
Fig 5H Dist. Travelled (Release, 200)	Kcng4 vs Pcp2	15 vs 19	0.96
Fig 5H Dist. Travelled (Release, 200)	Kcng4 vs Camk2	15 vs 8	<0.001
Fig 5H Dist. Travelled (Release, 200)	Pcp2 vs Camk2	19 vs 8	<0.001
Fig 5H Dist. Travelled (Release, 500)	Kcng4 vs Pcp2	15 vs 19	0.88
Fig 5H Dist. Travelled (Release, 500)	Kcng4 vs Camk2	15 vs 8	<0.001
Fig 5H Dist. Travelled (Release, 500)	Pcp2 vs Camk2	19 vs 8	<0.001
Fig 5H Dist. Travelled (Release, 1000)	Kcng4 vs Pcp2	15 vs 19	0.86
Fig 5H Dist. Travelled (Release, 1000)	Kcng4 vs Camk2	15 vs 8	<0.001
Fig 5H Dist. Travelled (Release, 1000)	Pcp2 vs Camk2	19 vs 8	<0.001
Fig 5H Dist. Travelled (Recover, 200)	Kcng4 vs Pcp2	15 vs 19	0.43
Fig 5H Dist. Travelled (Recover, 200)	Kcng4 vs Camk2	15 vs 8	>0.99
Fig 5H Dist. Travelled (Recover, 200)	Pcp2 vs Camk2	19 vs 8	0.83
Fig 5H Dist. Travelled (Recover, 500)	Kcng4 vs Pcp2	15 vs 19	0.01
Fig 5H Dist. Travelled (Recover, 500)	Kcng4 vs Camk2	15 vs 8	0.99
Fig 5H Dist. Travelled (Recover, 500)	Pcp2 vs Camk2	19 vs 8	0.60
Fig 5H Dist. Travelled (Recover, 1000)	Kcng4 vs Pcp2	15 vs 19	0.03
Fig 5H Dist. Travelled (Recover, 1000)	Kcng4 vs Camk2	15 vs 8	>0.99
Fig 5H Dist. Travelled (Recover, 1000)	Pcp2 vs Camk2	19 vs 8	0.17
Fig 6D (NT 0.1 Hz)	Kcng4 vs Pcp2	15 vs 19	0.0167
Fig 6D (NT 0.2 Hz)	Kcng4 vs Pcp2	15 vs 19	0.9985
Fig 6D (NT 0.4 Hz)	Kcng4 vs Pcp2	15 vs 19	>0.9999
Fig 6D (NT 0.6 Hz)	Kcng4 vs Pcp2	15 vs 19	>0.9999
Fig 6D (NT 0.8 Hz)	Kcng4 vs Pcp2	15 vs 19	>0.9999
Fig 6D (NT 1.0 Hz)	Kcng4 vs Pcp2	15 vs 19	>0.9999
Fig 6D (TN 0.1 Hz)	Kcng4 vs Pcp2	15 vs 19	0.0002
Fig 6D (TN 0.2 Hz)	Kcng4 vs Pcp2	15 vs 19	0.7168
Fig 6D (TN 0.4 Hz)	Kcng4 vs Pcp2	15 vs 19	>0.9999
Fig 6D (TN 0.6 Hz)	Kcng4 vs Pcp2	15 vs 19	>0.9999

Fig 6D (TN 0.8 Hz)	Kcng4 vs Pcp2	15 vs 19	>0.9999
Fig 6D (TN 1.0 Hz)	Kcng4 vs Pcp2	15 vs 19	>0.9999
Fig 6E (NT 0.1 Hz)	Kcng4 vs Pcp2	15 vs 19	<0.0001
Fig 6E (NT 0.2 Hz)	Kcng4 vs Pcp2	15 vs 19	0.9290
Fig 6E (NT 0.4 Hz)	Kcng4 vs Pcp2	15 vs 19	>0.9999
Fig 6E (NT 0.6 Hz)	Kcng4 vs Pcp2	15 vs 19	>0.9999
Fig 6E (NT 0.8 Hz)	Kcng4 vs Pcp2	15 vs 19	>0.9999
Fig 6E (NT 1.0 Hz)	Kcng4 vs Pcp2	15 vs 19	>0.9999
Fig 6E (TN 0.1 Hz)	Kcng4 vs Pcp2	15 vs 19	<0.0001
Fig 6E (TN 0.2 Hz)	Kcng4 vs Pcp2	15 vs 19	0.5923
Fig 6E (TN 0.4 Hz)	Kcng4 vs Pcp2	15 vs 19	>0.9999
Fig 6E (TN 0.6 Hz)	Kcng4 vs Pcp2	15 vs 19	>0.9999
Fig 6E (TN 0.8 Hz)	Kcng4 vs Pcp2	15 vs 19	>0.9999
Fig 6E (TN 1.0 Hz)	Kcng4 vs Pcp2	15 vs 19	>0.9999
Fig 6F (NT 0.1 Hz)	Kcng4 vs Pcp2	15 vs 19	0.0333
Fig 6F (NT 0.2 Hz)	Kcng4 vs Pcp2	15 vs 19	0.9692
Fig 6F (NT 0.4 Hz)	Kcng4 vs Pcp2	15 vs 19	>0.9999
Fig 6F (NT 0.6 Hz)	Kcng4 vs Pcp2	15 vs 19	>0.9999
Fig 6F (NT 0.8 Hz)	Kcng4 vs Pcp2	15 vs 19	>0.9999
Fig 6F (NT 1.0 Hz)	Kcng4 vs Pcp2	15 vs 19	>0.9999
Fig 6F (TN 0.1 Hz)	Kcng4 vs Pcp2	15 vs 19	0.0056
Fig 6F (TN 0.2 Hz)	Kcng4 vs Pcp2	15 vs 19	0.9867
Fig 6F (TN 0.4 Hz)	Kcng4 vs Pcp2	15 vs 19	>0.9999
Fig 6F (TN 0.6 Hz)	Kcng4 vs Pcp2	15 vs 19	>0.9999
Fig 6F (TN 0.8 Hz)	Kcng4 vs Pcp2	15 vs 19	>0.9999
Fig 6F (TN 1.0 Hz)	Kcng4 vs Pcp2	15 vs 19	>0.9999

144
